# Integrating Microbial Indicators from High-Throughput Sequencing into Soil Quality Index: A Case Study in a Restored Mining Area

**DOI:** 10.3390/microorganisms14092009

**Published:** 2026-09-10

**Authors:** Zhengjun Feng, Chaolong Ma, Shengxin Yan, Wenhui Liu, Peiyin Li, Yan Zou, Dashdorj Munkhbat, Huiping Song

**Affiliations:** 1 Institute of Resources and Environmental Engineering, Shanxi University, Taiyuan 030006, China; fzj@sxu.edu.cn (Z.F.);; 2 Engineering Research Center of Resource Efficiency Enhancing and Carbon Emission Reduction in Yellow River Basin, Ministry of Education of the People’s Republic of China, Taiyuan 030006, China; 3 Shanxi Qinghuan Nengchuang Environmental Protection Technology Company Limited, Taiyuan 030006, China; 4 School of Engineering and Technology, Mongolian University of Life Sciences, Ulaanbaatar 17029, Mongolia

**Keywords:** soil quality index, high-throughput sequencing, microbial communities

## Abstract

The Soil Quality Index (SQI) is a vital tool for evaluating soil quality; however, traditional approaches seldom integrate microbial data from high-throughput sequencing—commonly used to characterize soil microbial communities—into the SQI framework. This study enhances the SQI by incorporating microbial indicators derived from high-throughput sequencing, establishing a more comprehensive evaluation system. We collected soil samples from mining areas and analyzed their fundamental physicochemical properties and microbial indicators. Four SQI models were constructed using different indicator sets: (1) only physicochemical properties (T-SQI); (2) physicochemical properties and bacterial α-diversity (α-SQI); (3) physicochemical properties, α-diversity, and relative abundances of the top five abundant bacteria (αMA-SQI); and (4) physicochemical properties, α-diversity, and relative abundances of the top five bacteria based on LDA scores (αBM-SQI). Results demonstrated that integrating multi-level microbial indicators improved the rationality of soil quality rankings and significantly strengthened correlations with α-diversity. Gemmatimonadota was consistently selected in the Minimum Data Set (MDS) for both αMA-SQI and αBM-SQI, highlighting its ecological importance. Statistically, microbial indicators at the order and family levels were most suitable for inclusion in the MDS, as their results deviated least from the total dataset. In conclusion, incorporating microbial diversity across taxonomic levels refines the SQI, enabling a more accurate and holistic assessment of soil health.

## 1. Introduction

Soil quality is defined as the capacity of soil to function within ecosystem boundaries, sustain biological productivity, maintain environmental quality, and support plant and animal health [1]. Globally, soil quality has experienced significant decline due to a combination of anthropogenic and natural factors [2,3]. Mining activities, in particular, exacerbate this degradation by not only causing ecological and resource depletion but also inducing various geological hazards [4,5]. To enable a holistic evaluation of soil quality, the Soil Quality Index (SQI) has been developed as an integrative metric that synthesizes key soil properties into a single comprehensive indicator, widely applied in both scientific and practical contexts. The SQI should be based on a minimum data set (MDS), comprising a select number of interrelated parameters that quantitatively reflect essential soil functions [6]. Due to the high heterogeneity and complexity of soil ecosystems, individual physicochemical or biological indicators are often insufficient to fully capture soil health status.

The construction of the SQI generally involves two critical steps: first, the scientific selection of representative evaluation indicators based on soil functions and ecosystem services, and second, the extraction of a minimum data set (MDS) from these initial indicators through Principal Component Analysis (PCA) to enhance the reliability and practicality of the assessment [7]. Among various methods proposed for computing a comprehensive soil quality index, the approach introduced by Andrews et al. [8] remains the most widely adopted. This method utilizes PCA to identify the MDS and models the underlying objective function via continuous multivariate regression. Subsequently, the selected indicators are normalized and aggregated using a weighted additive index (WAI), whereby each indicator is assigned a weight derived from the PCA results before being summed to yield a final quality score for each sampling unit.

In the early development of soil quality indices, evaluation criteria were predominantly centered on physicochemical properties. Conventional physical indicators included porosity, water content, and bulk density, while chemical indicators encompassed organic matter, total and available nutrients, pH, and electrical conductivity. However, as understanding of soil ecosystems advanced, the significance of microbial communities as vital indicators of soil quality has gained increasing recognition [9]. Soil microorganisms play integral roles in nutrient cycling, soil formation, and plant development [10], and are now recognized as sensitive markers of land degradation and ecological restoration success. Microbial activities contribute to enhanced soil structural stability [11], facilitate the formation and stabilization of soil aggregates, and reduce erosive particle loss [12]. Moreover, monitoring microbial community composition and activity offers a more holistic reflection of soil health, as microorganisms respond more rapidly to environmental changes than do physical or chemical properties [13,14]. Microbial biomass and its relative abundance are strongly correlated with soil fertility levels, providing critical insights into overall soil health and functionality [15,16].

With the continuous advancement of detection technologies, researchers have increasingly incorporated biological indicators into the calculation of the Soil Quality Index (SQI) [17]. These indicators primarily include microbial biomass, soil respiration, and enzyme activities. Their integration holds significant potential for developing a more comprehensive and scientifically robust system for assessing soil health. High-throughput sequencing (HTS) technology has become instrumental in elucidating the taxonomic composition, genetic diversity, and functional attributes of soil microbial communities [18]. Soils can harbor up to approximately 10 billion microbial cells per gram [19], with microbial abundance and diversity varying considerably across soil types, seasons, and depth profiles. HTS provides a comprehensive and detailed perspective on the interactions between soil microorganisms and ecological conditions [20,21], yielding rich data on microbial taxonomy, community structure, functional potential, and genetic diversity. These insights offer a solid foundation for the rational utilization and management of soil resources. Currently, most SQI studies focus predominantly on physicochemical properties, while microbial aspects are often restricted to broad measures such as soil respiration, enzyme activity, and microbial biomass. Given the critical roles microbes play in soil functioning, integrating HTS-derived microbial data into the established SQI framework could lead to a more holistic and accurate assessment of soil quality.

In summary, this study targeted a mining area in Taiyuan for soil sampling and incorporated microbial indicators into the Soil Quality Index (SQI) to establish a novel evaluation framework. The main research objectives include: (1) determining the physicochemical properties and microbial indicators of the collected soil samples; (2) assessing the scientific validity and rationality of different methodologies for integrating microbial indicators by comparing their influences on SQI outcomes; and (3) identifying relatively optimal computational approaches for evaluating soil quality.

## 2. Materials and Methods

### 2.1. Field Site

This study was carried out in the Yuquan Mountain area, located within the Xishan mining region of Taiyuan City, Shanxi Province, China. The specific sampling site is situated to the west of Geliaogou Village, administered by the Dongshe Street Office in Wanberlin District, Taiyuan (geographical coordinates: 37°90′ N, 112°43′ E). Covering an area of approximately 13 km^2^, Yuquan Mountain has a historical legacy of coal mining. In response to severe land degradation, ecological restoration initiatives were launched in the area beginning in 2009.

Yuquan Mountain lies within the Fen River watershed, part of the larger Yellow River Basin. The region experiences a warm-temperate continental monsoon climate with arid characteristics, featuring ample sunshine, distinct seasonal variations, and considerable diurnal temperature fluctuations. According to meteorological station records, the mean annual temperature is 9.5 °C, with extreme temperatures ranging from −27.5 °C to 39.4 °C. The average annual precipitation is 495.9 mm, with approximately 60% of the total rainfall occurring between July and September. The mean annual evaporation is 1849.3 mm, and the average wind speed is 2.5 m/s. The frost season typically commences in early October.

### 2.2. Soil Sampling

On 28 October 2023, 24 sampling sites were established in the Yuquan Mountain area of Taiyuan, Shanxi Province, representing different restoration durations, restoration practices, and vegetation types. At each sampling site, three parallel 1 m × 1 m quadrats were established along a line transect, resulting in 72 quadrats in total. Within each quadrat, soil was collected from three sampling points at a depth of 0–10 cm, yielding 216 field-collected soil subsamples across the 24 sampling sites.

To obtain a representative composite sample for each sampling site, the soil collected from the three parallel quadrats within the same sampling site was thoroughly mixed and homogenized. Thus, the 216 field-collected subsamples were composited to obtain 24 site-level composite soil samples, corresponding to sampling sites YQ1–YQ24. These 24 site-level composite samples were used for subsequent soil physicochemical and microbial analyses (Table 1).

### 2.3. Soil Analyses

Soil pH and electrical conductivity (EC) were determined using the electrode potential method and a conductivity meter, respectively. Soil organic matter (SOM) content was quantified via the potassium dichromate oxidation method with external heating. Total nitrogen (TN) was analyzed using an elemental analyzer, and alkaline-hydrolyzable nitrogen (AN) was measured by the alkaline diffusion method. Total phosphorus (TP) and available phosphorus (AP) were both assessed by molybdenum–antimony anti-colorimetry. Available potassium (AK) was extracted with 1 mol/L ammonium acetate (NH_4_OAc) and quantified by flame photometry.

### 2.4. DNA Extraction and 16S rRNA Gene Amplicon Sequencing

High-throughput sequencing of the 16S rRNA gene was conducted by Personalbio Co., Ltd. (Shanghai, China).

Soil microbial genomic DNA was extracted from soil subsamples stored at −80 °C using the OMEGA Soil DNA Kit (M5635-02; Omega Bio-Tek, Norcross, GA, USA) according to the manufacturer’s instructions. The concentration and quality of the extracted DNA were assessed using a NanoDrop NC2000 spectrophotometer (Thermo Fisher Scientific, Waltham, MA, USA) and agarose gel electrophoresis, respectively. The bacterial 16S rRNA gene V3–V4 hypervariable region was amplified using the primer pair 338F (5′-ACTCCTACGGGAGGCAGCA-3′) and 806R (5′-GGACTACHVGGGTWTCTAAT-3′). PCR amplification was conducted according to the standard library-construction protocol provided by Shanghai Personal Biotechnology Co., Ltd. (Shanghai, China). Purified amplicons were pooled and sequenced on an Illumina NovaSeq platform using paired-end 2 × 250 bp sequencing. An average of approximately 51,000 high-quality, non-chimeric reads was obtained per sample, and Good’s coverage exceeded 98% for all samples.

Bioinformatic analyses were conducted using QIIME 2 (v2019.4) through the GenesCloud platform. Raw sequence data were quality filtered and denoised using the DADA2 algorithm, which was used to infer amplicon sequence variants (ASVs) rather than conventional operational taxonomic units (OTUs). The resulting ASV table was rarefied to 95% of the minimum sequencing depth across samples. Taxonomic assignment of ASVs was performed using the naïve Bayes classifier implemented in the QIIME 2 feature-classifier plugin against the SILVA database (Release 138). The resulting taxonomic profiles were used for downstream analyses of bacterial community composition and diversity (Table 2).

### 2.5. Soil Quality Index

For PCA-based MDS selection and SQI construction, the sampling site was defined as the analytical unit. The three parallel quadrats established within each sampling site and the three soil sampling points within each quadrat were treated as spatial subsampling units rather than independent observations for the SQI analysis. Soil collected from the three quadrats within the same sampling site was thoroughly mixed and homogenized to form one site-level composite sample. Therefore, the spatial variability represented by the subsamples was incorporated during the composite sampling process, and no separate subsample-level observations from the same site were entered into the PCA or SQI calculations. The final analytical dataset consisted of 24 site-level composite observations (YQ1–YQ24), which were used for PCA-based MDS selection, Pearson correlation analysis, indicator scoring, SQI calculation, and ranking comparisons.

The Soil Quality Index (SQI) is widely employed to assess soil quality when a Minimum Data Set (MDS) is available, allowing researchers to identify the most representative soil indicators and minimize data redundancy [22]. The computation of SQI generally involves three key steps: (1) selection of the MDS; (2) scoring of the MDS indicators; and (3) integration of scores into a composite SQI value. To identify the most representative indicators for the MDS, Principal Component Analysis (PCA) coupled with Pearson correlation analysis is commonly applied. According to established criteria [23]. only principal components (PCs) with eigenvalues ≥1 that account for more than 5% of the total variance are considered. Within each eligible PC, factors with absolute loadings ranking within the top 10% are identified as important indicators. When multiple indicators are retained within one PC, Pearson correlation analysis is used to evaluate redundancy. If a strong correlation exists (i.e., correlation coefficient *r* > 0.6), only the variable with the highest absolute loading is retained. Once the MDS is established, a non-linear scoring function—often an S-shaped curve (Equation (1))—is applied to transform each indicator value into a unitless score between 0 and 1.

(1) $$S=a∕{\left[1+\left(x∕x_{0}\right)\right]}^{b}$$

where S represents the score of the soil indicator, *a* denotes the maximum score value (set to *a* = 1), x is the measured value of the indicator, *x*_0_ is the mean value of that indicator across the dataset, and *b* is the slope coefficient of the equation. The slope *b* is assigned a value of −2.5 for indicators following a “more is better” trend and +2.5 for those following a “less is better” trend. The final *SQI* is computed by integrating the scores and their respective weighting values according to Equation (2):

(2) $$SQI=\sum_{i=1}^{n}S_{i}\cdot W_{i}$$

where $W_{i}$ represents the weight of the soil indicator derived from the principal component analysis (PCA), *S_i_* denotes the score of the indicator obtained from Equation (1), and *n* corresponds to the number of indicators included in the minimum data set (MDS).

The integration of microbial indicators into a novel evaluation framework presents a challenge due to the high dimensionality of microbial data, particularly those derived from high-throughput sequencing. Key microbial metrics include α-diversity, gene abundance, and taxonomic composition. Given the impracticality of incorporating all available indicators, it is essential to identify the most rational and comprehensive subset. Three methodological approaches have been proposed: (1) α-Diversity only: α-Diversity effectively captures the richness and evenness of soil microbial communities and serves as a fundamental indicator of soil health [24]. (2) α-Diversity combined with gene abundance: In addition to α-diversity, incorporating the top five most abundant genes across taxonomic levels can reflect the expression levels of specific functional genes [7], thereby providing insight into the functional potential of soil microbial communities. (3) Selection of dominant species: Dominant taxa often play pivotal roles in soil ecosystems and exert considerable influence on soil functioning. Thus, indicators based on dominant species may more accurately represent the status of the soil microbial community [25].

### 2.6. Statistical Analysis

The original soil property data were processed using Microsoft Excel 2020. Descriptive statistics (including means and standard errors), principal component analysis (PCA), and post-hoc comparisons using Duncan’s multiple range test (with significance defined at *p* < 0.05) were performed with IBM SPSS Statistics 26. Pearson correlation analysis was applied to evaluate relationships among soil indicators. All figures and graphical representations were generated using Origin 2021. Analysis of soil microbial community data—including species composition, α- and β-diversity, and differential species analysis—was conducted on the Genescloud platform (Table 3).

## 3. Results

### 3.1. Soil Indicators

**Figure 1:**
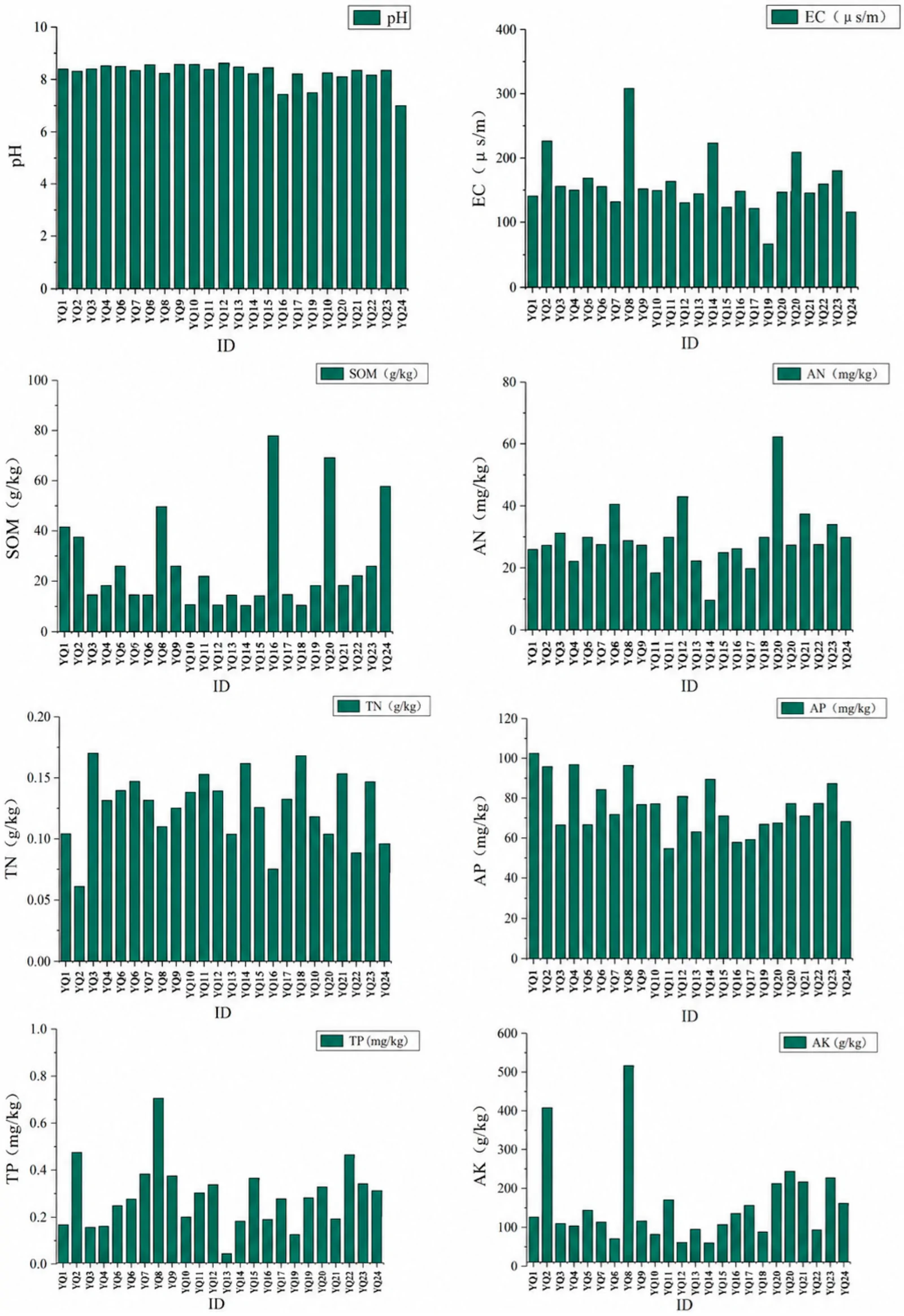
Soil physicochemical indicators of the investigated soil samples. Bar charts of eight soil physicochemical indicators including pH, electrical conductivity (EC), soil organic matter (SOM), alkaline-hydrolyzable nitrogen (AN), total nitrogen (TN), available phosphorus (AP), total phosphorus (TP), and available potassium (AK) for samples YQ1–YQ24. The x-axis represents sample identifiers, and the y-axis shows measured values for each soil parameter with corresponding units. Each subplot corresponds to one individual soil indicator. The figure demonstrates the variation characteristics of soil physicochemical properties across all soil samples.

The soil pH across different restoration types in the study area ranged from 7.21 to 8.83, indicating slightly alkaline conditions overall [26]. Notably, the pH of sample 24 was significantly lower than that of all other samples. Electrical conductivity (EC) values were generally below 300 μS/cm, with the exception of sample 8, which exceeded this threshold, suggesting mild salinization [27]. The content of alkaline-hydrolyzable nitrogen (AN) ranged from 9.33 to 58.33 mg/kg, which is generally low. Sample YQ19 showed the highest AN content (58.33 mg/kg), significantly exceeding other samples, while YQ14 had the lowest value (9.33 mg/kg). In contrast, available phosphorus (AP) was relatively high, with a minimum value of 54.02 mg/kg. Available potassium (AK) varied considerably among the samples: YQ8 and YQ2 contained 517.00 mg/kg and 418.33 mg/kg, respectively, significantly higher than other samples, which ranged between 47.67 and 233.00 mg/kg. Total nitrogen (TN) was consistently low across all samples, ranging from 0.07 to 0.18 g/kg. Similarly, total phosphorus (TP) remained below 1 g/kg, indicating very low levels. Soil organic matter (SOM) exceeded 10 g/kg in all samples, with the highest value of 68.79 g/kg observed in sample YQ16 (Figure 1).

### 3.2. Microbiological

**Figure 2:**
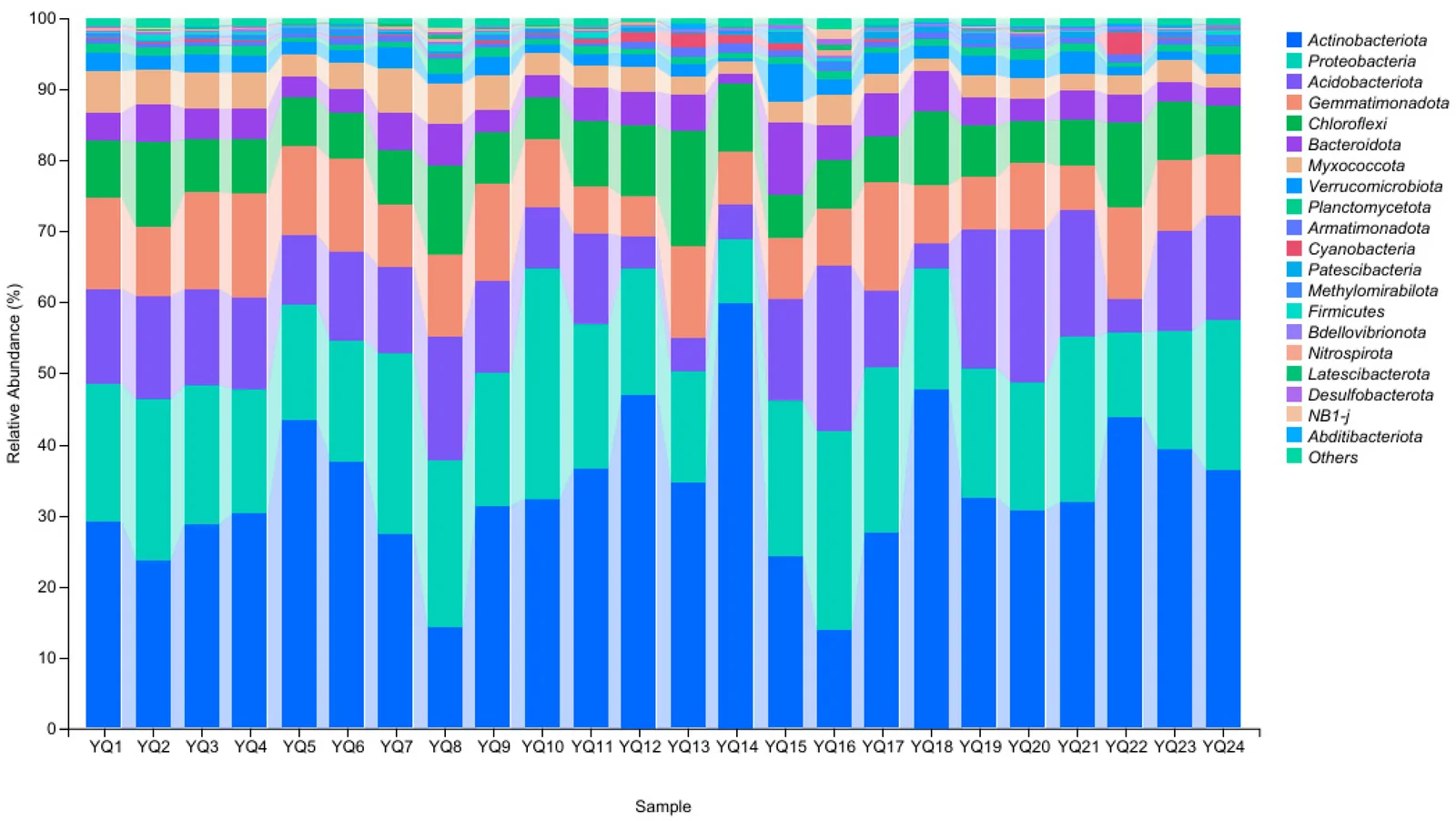
Composition and distribution of soil microorganisms in different treatment areas at the phylum level. Relative abundance of soil microbial taxa at the phylum level across different samples (YQ1–YQ24). The x-axis represents sample identifiers, and the y-axis shows relative abundance (%). Distinct colors correspond to major phyla such as Actinobacteriota, Proteobacteria, Acidobacteriota, etc. The figure illustrates the compositional differences in soil microbial communities among the various treatment zones.

Figure 2 displays the compositional distribution of microbial communities at the phylum level across the sampled sites. Marked differences in the relative abundance of major phyla were observed among the treatment areas. The dominant phyla consisted of Actinobacteria, Proteobacteria, Acidobacteria, Gemmatimonadetes, and Chloroflexi, collectively accounting for approximately 80% of the total microbial community.

Analysis of the dominant phyla revealed that sample YQ14 exhibited the highest relative abundance of Actinobacteria (59.80%), significantly surpassing all other samples. Conversely, the relative abundances of Proteobacteria, Acidobacteria, and Gemmatimonadetes were lower in YQ14 compared to other sites. In contrast, sample YQ16 showed the lowest proportion of Actinobacteria (13.70%) but the highest relative abundance of Proteobacteria (28.10%) among all samples. In samples YQ2 and YQ8, the five dominant phyla constituted 82.65% and 79.31% of the community, respectively. Previous studies [28] have indicated that Actinobacteria can form symbiotic relationships with certain plants, suggesting that the variation in microbial abundance may be linked to differences in vegetation across sampling sites—a relationship warranting further investigation (Table 4).

**Figure 3:**
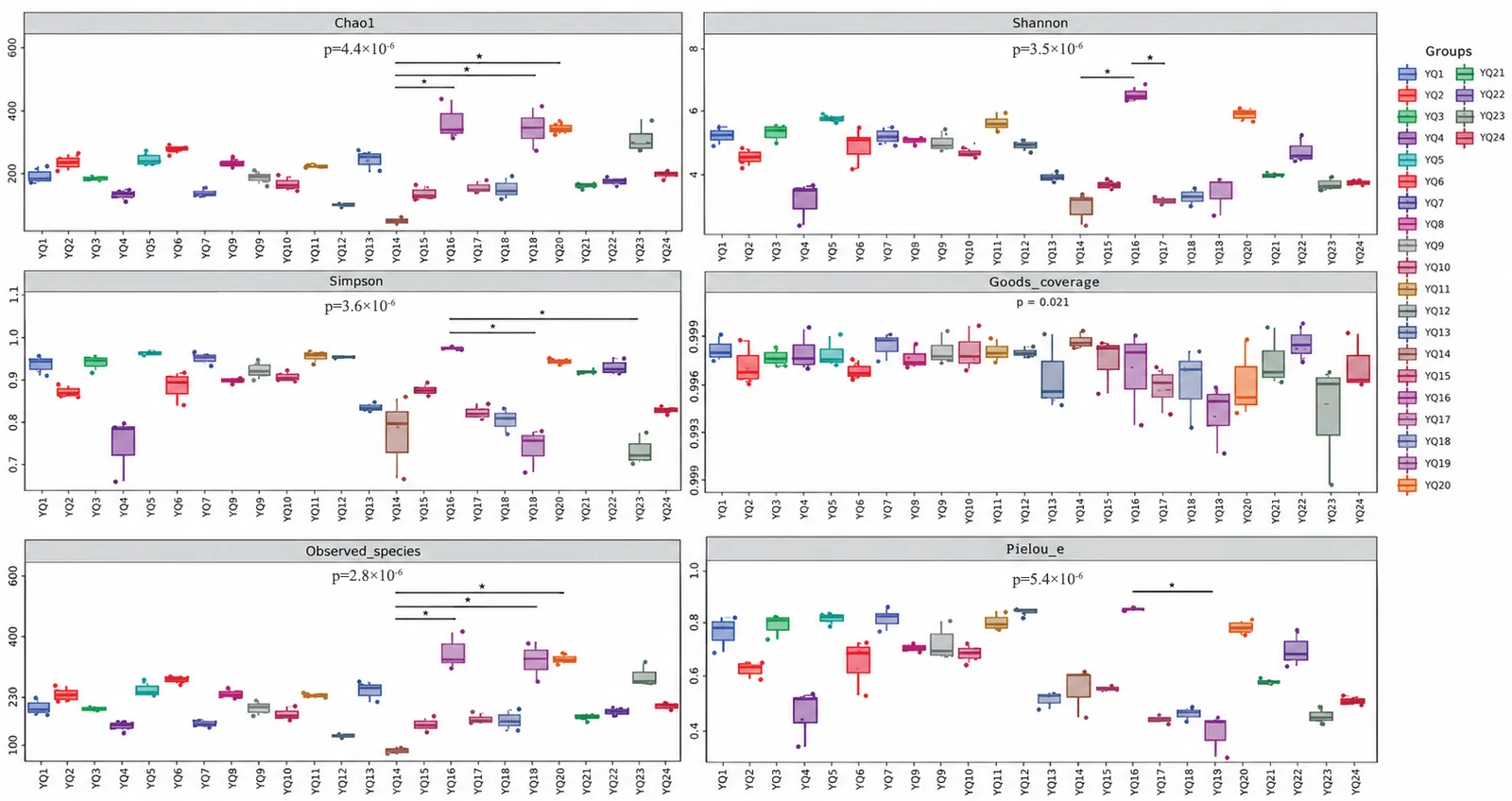
Microbial Community α-Diversity in Different Sampling Samples. Box plots of α-diversity metrics (Chao1, Shannon, Simpson, Good’s coverage, Observed species, and Pielou’s evenness) for microbial communities across the different sampling groups. Each box displays the median, interquartile range, and whiskers (1.5 × IQR), with outliers shown as individual points. Statistical comparisons are indicated by *p*-values; significant differences (*p* < 0.05) are observed for all indices except Good’s coverage (*p* = 0.24), which shows no significant variation among groups. * *p* < 0.05.

In this study, species richness was evaluated using the Chao1 and Observed species indices, both of which are positively correlated with richness. Species diversity was assessed using the Simpson and Shannon indices, with higher values indicating greater diversity. Sample coverage was estimated using Good’s coverage index (Figure 3).

Both the Chao1 and Observed species indices were lower in sample YQ14 than in other samples, suggesting that environmental conditions may have adversely affected microbial richness at this site. The coverage values exceeded 98% across all samples, indicating that the sequencing depth sufficiently captured the microbial diversity present. With regard to diversity, the Shannon and Simpson indices were significantly lower in samples YQ4, YQ14, and YQ19, implying reduced microbial diversity and ecosystem stability in these areas.

### 3.3. Selection of Indicators

In this study, the assessment of soil physicochemical properties in the 0–10 cm layer was based solely on pH, EC, AN, TN, AP, TP, AK, SOM, and Na. Principal component analysis (PCA) extracted three principal components with eigenvalues ≥1. In PC-1, EC, AK, and Na exhibited high loading values. Due to significant correlations among these variables (EC–AK: *r* = 0.734; EC–Na: *r* = 0.820; both exceeding the threshold of |*r*| > 0.6), only EC was retained. In PC-2, pH and SOM demonstrated high loadings, and their absolute correlation value reached 0.630, also above 0.6; thus, only pH was selected. In PC-3, AN displayed a notably high weighting. Consequently, EC, pH, and AN were identified as the optimal set of physicochemical indicators for evaluating soil quality in this system (Table 5).

Following the incorporation of α-diversity indices, principal component analysis yielded four principal components with eigenvalues ≥1. In PC-1, high loadings were observed for Observed_species, Faith_pd, Shannon, Chao1, and Pielou_e. Due to significant intercorrelations among these indices and based on the highest factor loading, only Observed_species was retained. In PC-2, both EC and Na exhibited high weights, with a correlation coefficient of 0.820 between them (exceeding the 0.6 threshold), leading to the retention of EC. PC-3 was dominated by pH, while PC-4 was characterized by AN. Consequently, Observed_species, EC, pH, and AN were identified as the optimal indicator set for evaluating soil quality when integrating both soil physicochemical properties and bacterial α-diversity.

For the analysis incorporating both soil physicochemical properties and microbial indicators, we conducted evaluations across six taxonomic levels: phylum, class, order, family, genus, and species (Table 6).

At the phylum level, principal component analysis yielded five components with eigenvalues ≥1. In PC-1, high loadings were observed for Faith_pd, Acidobacteriota, p_Actinobacteriota, Chao1, Shannon, Pielou_e, and Actinobacteriota. Due to significant intercorrelations among these eight indices and based on the highest factor loading, only Faith_pd was retained. In PC-2, both EC and Chloroflexi exhibited dominant loadings, with a correlation coefficient of 0.292 between them—below the 0.6 threshold—resulting in the retention of both indicators. PC-3 was characterized by the highest weight for p_Proteobacteria, while PC-4 was dominated by Gemmatimonadaceae. PC-5 showed the strongest weighting for AN. Consequently, Faith_pd, EC, Chloroflexi, p_Proteobacteria, Gemmatimonadaceae, and AN were identified as the optimal indicator set for assessing soil quality at the phylum level.

At the class level, five principal components were extracted with eigenvalues ≥1. PC-1 exhibited high loadings for c_Vicinamibacteria, Chao1, Thermoleophilia, and Shannon. Due to significant correlations among these four indices, only c_Vicinamibacteria—displaying the highest loading—was retained. PC-2 was characterized by the dominant loading of c_Blastocatellia, while PC-3 was represented by c_Alphaproteobacteria. PC-4 and PC-5 exhibited the strongest loadings for AN and Gemmatimonadetes, respectively. Therefore, c_Vicinamibacteria, c_Blastocatellia, c_Alphaproteobacteria, AN, and Gemmatimonadetes were identified as the optimal indicators for assessing soil quality at the class level.

At the order level, five principal components were extracted with eigenvalues ≥1. PC-1 exhibited high loadings for Vicinamibacterales, Shannon, Faith_pd, Pielou_e, o_Vicinamibacterales, and Solirubrobacterales. Due to significant intercorrelations among these six indices, only Vicinamibacterales was retained based on its highest loading value. PC-2 was dominated by o_Pyrinomonadales, while PC-3 and PC-4 showed the strongest loadings for AK and Gemmatimonadales, respectively. PC-5 demonstrated high weights for both Burkholderiales and AN, with a correlation coefficient of 0.222 between them—below the 0.6 threshold—resulting in the retention of both indicators. Consequently, Vicinamibacterales, o_Pyrinomonadales, AK, Gemmatimonadales, Burkholderiales, and AN were identified as the optimal indicator set for soil quality assessment at the order level.

At the family level, four principal components met the extraction criteria. PC-1 displayed high loadings for Vicinamibacteraceae, Chao1, Shannon, Faith_pd, f_Vicinamibacteraceae, Pielou_e, and Solirubrobacteraceae. Given the significant correlations among these seven indices, only Vicinamibacteraceae was retained. PC-2 was characterized by the highest weighting for EC, while PC-3 was represented by f_Pyrinomonadaceae. PC-4 exhibited dominant loadings for AN, Gemmatimonadaceae, and Nocardioidaceae. The correlation coefficients between these three indicators (Gemmatimonadaceae-Nocardioidaceae: −0.134; Gemmatimonadaceae-AN: −0.196) all had absolute values below 0.6, leading to the retention of all three. Thus, Vicinamibacteraceae, EC, f_Pyrinomonadaceae, Gemmatimonadaceae, Nocardioidaceae, and AN comprise the optimal indicator set for evaluating soil quality at the family level.

At the genus level, four principal components were extracted with eigenvalues ≥1. PC-1 exhibited high loadings for Vicinamibacteraceae, Faith_pd, Chao1, g_Vicinamibacteraceae, and Shannon. Due to significant intercorrelations among these five indices, only Vicinamibacteraceae was retained based on its highest loading value. PC-2 demonstrated dominant loadings for both g_RB41 and species, with a correlation coefficient of |*r*| = 0.417 between them—below the 0.6 threshold—resulting in the retention of both indicators. PC-3 was characterized by the highest weighting for g_Allorhizobium-Neorhizobium-Pararhizobium-Rhizobium, while PC-4 was dominated by AN. Therefore, Vicinamibacteraceae, g_RB41, species, g_Allorhizobium-Neorhizobium-Pararhizobium-Rhizobium, and AN were identified as the optimal indicator set for assessing soil quality at the genus level.

At the species level, four principal components met the extraction criteria. PC-1 displayed high loadings for Chao1, Faith_pd, Shannon, and Pielou_e. Given the significant correlations among these four indices, only Chao1 was retained. PC-2 exhibited the strongest weighting for Archangium_gephyra, while PC-3 was dominated by Sporichthya_polymorpha. PC-4 showed the highest loading for AN. Consequently, Chao1, Archangium_gephyra, Sporichthya_polymorpha, and AN comprise the optimal indicator set for evaluating soil quality at the species level.

Furthermore, when evaluating soil quality based exclusively on dominant taxa and physicochemical properties, the Soil Quality Index (SQI) was computed at four taxonomic levels: phylum, class, order, and family. At the phylum level, EC, species richness, Observed_species, Proteobacteria, and Gemmatimonadota were identified as optimal indicators for assessing soil quality. At the class level, EC, AN, Observed_species, and Alphaproteobacteria were selected as the most representative indicators. For the order level, AN, Na, Chao1, and Gemmatimonadales comprised the optimal indicator set. At the family level, AN, TN, Na, Faith_pd, Gemmatimonadaceae, Sphingomonadaceae, and the taxon designated 67-14 were retained as key indicators. Collectively, these curated sets of indicators provide a comprehensive representation of soil health across different taxonomic resolutions.

### 3.4. Comparison of SQI

The T-SQI values across sampling sites ranged from 0.29 to 2.31, with the maximum observed at YQ19 and the minimum at YQ14. However, the incorporation of microbial indicators significantly altered SQI values at all taxonomic levels, resulting in substantial fluctuations in the overall site rankings.

Under the T-SQI framework, site YQ5 exhibited the highest value while YQ14 showed the lowest. Following the integration of microbial indicators, substantial changes occurred in site rankings. In both the αMA-SQI and α-SQI assessments, YQ14 consistently remained the lowest-ranked site. For α-SQI and αMA-SQI across phylum, class, order, family, and genus levels, YQ19 emerged as the top-ranked site. At the species level, however, YQ10 demonstrated the highest value. In the αBM-SQI analysis, YQ14 maintained the lowest values at both phylum and class levels, while the highest values were observed at YQ10 and YQ19, respectively. At the order and family levels, the maximum values shifted to YQ8 and YQ14, respectively, with YQ18 consistently showing the lowest values at both these taxonomic levels.

From the perspective of actual field conditions at the sampling sites, the ranking derived from T-SQI exhibited certain inconsistencies. The integration of microbial indicators, however, yielded a more ecologically meaningful ranking pattern. Specifically, plots YQ1-YQ12 with only one year of restoration would be expected to demonstrate lower SQI values, as extended restoration periods generally correlate with improved soil quality. This expected pattern was better reflected after microbial indicator incorporation.

Analysis of mean rankings revealed that classifications based on αMA-SQI at class, order, family, and genus levels, as well as αBM-SQI at class level, showed systematically lower average rankings compared to T-SQI after microbial indicator integration. The initially anomalous T-SQI rankings for bare land plots YQ14 and YQ22 were substantially improved and better aligned with field observations following microbial indicator inclusion. Similarly, YQ16 and YQ17—both ten-year restoration plots—demonstrated significantly enhanced rankings with microbial indicators, consistent with established research findings.

In contrast, YQ18 and YQ23—two-year artificial restoration plots of Pinus tabuliformis—exhibited overall ranking declines after microbial indicator integration. Notably, YQ19—an eight-year artificially restored plot containing Pinus tabuliformis and Rosa xanthina—consistently achieved top rankings across multiple calculations, indicating superior soil quality. Plots YQ20 and YQ21, despite their ten-year restoration history, initially showed relatively low rankings that improved substantially and stabilized at elevated levels following microbial indicator inclusion. YQ24—a historically aerial-seeded plot from the Republican era—demonstrated more ecologically reasonable rankings after microbial integration. However, the five-year restoration plots YQ13 and YQ15 continued to show comparatively low rankings across assessments, suggesting persistent underlying limitations.

**Figure 4:**
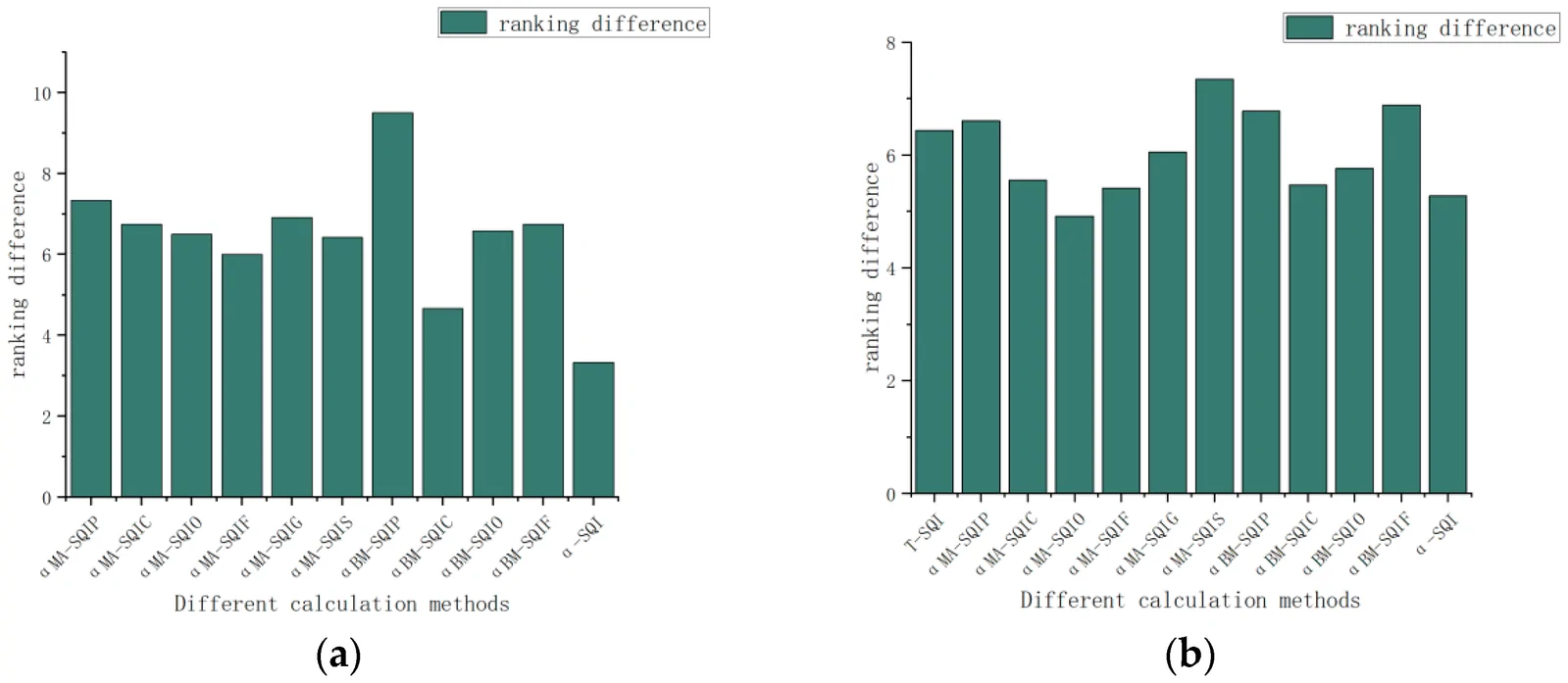
Different methods of comparing various computational results. Figure (**a**) illustrates the comparison of rankings between T-SQI and other computational methods under different calculation approaches. Figure (**b**) demonstrates the comparison of rankings among various computational methods themselves. T-SQI, αMA-SQIphyl, αMA-SQIcls, αMA-SQIord, αMA-SQIfam, αMA-SQIgen, αMA-SQIspp, αBM-SQIphyl, αBM-SQIcls, αBM-SQIord, αBM-SQIfam, α-SQI are different calculation methods.

Following the calculation of SQI across different taxonomic units, comparisons were made with the traditional T-SQI approach. As illustrated in Figure 4, the most substantial alterations occurred at the αMA-SQIphyl level, with an average rank shift of 9.50 positions per site. In comparison, the αBM-SQIcls exhibited the minimal average change (4.67 positions per site) among all taxonomic levels except for the α-SQI. The average rank changes for αMA-SQIcls, αMA-SQIord, and αMA-SQIfam were 6.75, 6.50, and 6.00 positions per site, respectively, with the αMA-SQIfam demonstrating a below-average shift relative to other levels.

Furthermore, SQI calculations derived from different taxonomic resolutions induced varying degrees of rank reorganization across sampling sites. Figure 4 illustrates the average rank change differences between T-SQI, αMA-SQI, and αBM-SQI rankings for each plot relative to other calculation methods. The mean ranking change across all taxonomic levels was 6.04 positions per site. Specifically, αMA-SQIord induced the most stable rankings with an average shift of merely 4.92 positions per site, while αMA-SQIspp provoked the most substantial reorganization, reaching an average of 7.34 positions per site.

## 4. Discussion

### 4.1. Restoration Age Alone Did Not Determine Soil Quality

Previous studies have shown that soil quality and soil microbial communities generally recover with increasing restoration or reclamation age [29,30,31,32]. In reclaimed mine soils, increasing reclamation age can promote soil development and gradual recovery of bacterial community structure and diversity [31,32]. However, the present study did not show a simple monotonic increase in SQI with restoration duration. Sites with longer restoration histories did not consistently receive higher rankings, indicating that restoration age alone was insufficient to explain the observed variation in soil quality.

This pattern is likely related to differences in vegetation composition, restoration type, and soil environmental conditions among the sampling sites. Soil quality is a multidimensional property involving interacting physicochemical and biological attributes, and these attributes may recover at different rates during restoration [33,34]. In the present study, YQ18 and YQ23 were both two-year artificial restoration sites dominated y *Pinus tabuliforis*, but neither consistently received high rankings after microbial information was incorporated. In contrast, YQ19, an eight-year artificially restored site wih *Pinus tabuliforis* ad *Rosa aciculais*, ranked first in several αMA-SQI formulations. Several 10-year restoration sites also showed relatively high rankings after microbial integration (Table 7). These differences suggest that restoration trajectories are influenced by the combined effects of restoration duration, vegetation characteristics, and soil development rather than by elapsed time alone.

YQ14 provides a clear example of the interaction between physicochemical and microbial conditions. This site had the lowest T-SQI value (0.29), the lowest AN concentration (9.33 mg kg^−1^), and relatively low bacterial richness and diversity. Its low Chao1 and Observed_species values and reduced Shannon and Simpson indices were consistent with its persistently low ranking in most microbial-integrated SQIs (Table 7 and Table 8). In contrast, YQ19 had a relatively high AN concentration (58.33 mg·kg^−1^) and ranked highly across several SQI formulations (Table 8). These contrasting patterns suggest that soil recovery in the studied mining area involved coordinated changes in nutrient availability and microbial community characteristics. Therefore, restoration age should be interpreted as an indicator of the restoration trajectory rather than as a sole determinant of soil quality.

### 4.2. Microbial Indicators Complemented Conventional Physicochemical Indicators

The MDS results demonstrate that microbial indicators complemented rather than replaced conventional physicochemical indicators. EC, pH, and AN constituted the MDS of T-SQI, while EC and/or AN remained in several microbial-integrated models (Table 6). The repeated selection of these variables indicates that basic soil chemical conditions and nutrient availability remained important dimensions of soil quality after microbial information was incorporated. This agrees with the multidimensional concept of soil quality, which emphasizes the need to combine complementary indicators representing different soil functions and biological processes [33,34].

Soil pH is particularly relevant to microbial community variation. It is widely recognized as an important environmental factor shaping bacterial community composition and diversity [35]. A global analysis of 942 soil samples demonstrated that soil pH strongly influenced bacterial diversity and the distribution of bacterial genera across different soil environments [35]. In reclaimed mine soils, pH and other physicochemical properties have likewise been associated with variation in bacterial community structure [32,36]. Therefore, the retention of pH in the T-SQI and α-SQI MDSs is consistent with its importance as an environmental factor affecting both soil conditions and bacterial communities.

AN was also repeatedly retained in the different MDSs (Table 6). As an indicator of soil nitrogen availability, AN represents an important component of soil fertility that cannot be fully replaced by microbial richness or taxonomic composition. The repeated selection of AN therefore indicates that microbial variables and physicochemical indicators provide complementary information. This interpretation is consistent with the proposed role of microbial indicators as complementary measures of soil condition rather than substitutes for conventional soil properties [34].

The effect of microbial integration was evident in the changes in site rankings (Table 7 and Table 8). T-SQI ranked YQ5 highest, whereas YQ19 ranked first in several αMA-SQI models. At the same time, YQ14 remained the lowest-ranked site in most microbial-integrated assessments. These changes indicate that microbial variables introduced an additional biological dimension into the SQI and altered the relative evaluation of sites according to differences in bacterial community characteristics.

The correlation analysis further supports this interpretation (Table 9). T-SQI showed relatively weak correlations with the major α-diversity indices, whereas several microbial-integrated SQIs showed stronger positive associations. For example, αBM-SQIphyl showed positive correlations with Chao1 (*r* = 0.703), Faith_pd (*r* = 0.658), Observed_species (*r* = 0.687), and Shannon (*r* = 0.686), while αBM-SQIord showed corresponding correlations of 0.680, 0.643, 0.674, and 0.648. Several αMA-SQI formulations also showed positive relationships with α-diversity. These results indicate that microbial-integrated SQIs incorporated information more closely associated with bacterial diversity than T-SQI.

However, these stronger associations should be interpreted as evidence of greater biological representation rather than direct proof of superior overall accuracy. Soil quality encompasses multiple dimensions, whereas α-diversity represents only one component of soil biological status [33,34]. Moreover, the correlations in Table 9 were calculated from the same dataset used for SQI construction and therefore represent internal consistency rather than independent validation. Additional measurements of microbial biomass, enzyme activity, soil respiration, plant productivity, or nutrient-cycling processes would provide stronger evidence for evaluating the functional performance of microbial-enhanced SQIs.

### 4.3. Taxonomic Resolution Affected SQI Ranking Stability

The results further demonstrated that taxonomic resolution influenced the extent to which microbial information changed SQI rankings (Figure 3). Among the αMA-SQI formulations, the largest average rank shift relative to T-SQI occurred at the phylum level (9.50 positions per site), whereas the order-level model showed a smaller shift of 4.92 positions per site. The family-level model also showed relatively stable rankings, with an average shift of 6.00 positions per site. In contrast, the species-level model produced comparatively large ranking changes.

These differences indicate that the taxonomic level used to represent microbial community composition can influence the stability of the resulting SQI. Broad phylum-level indicators integrate many taxa and may therefore provide relatively coarse information, whereas finer taxonomic levels retain more detailed community variation and may generate greater differences among sampling sites. The use of ASV-based inference in the present study provided high-resolution sequence information [37], but higher taxonomic resolution does not necessarily result in a more stable ecological indicator. Thus, the observed ranking differences may reflect a balance between the ecological information retained and the sensitivity of the indicator set to community variation.

**Figure 5:**
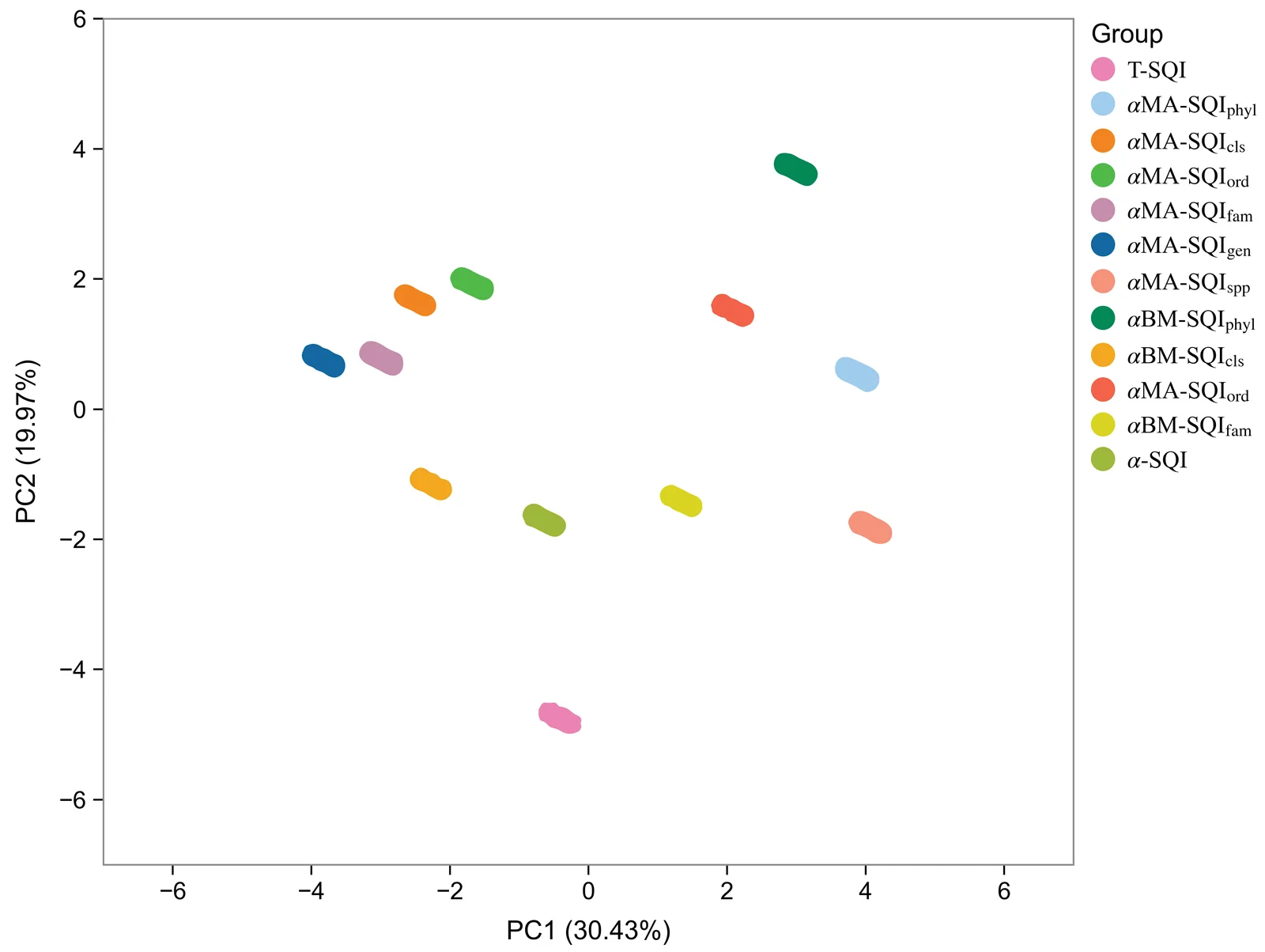
PCA samples with different calculation methods for ranking. Principal Component Analysis (PCA) scatter plot illustrating the clustering of samples obtained with different ranking calculation methods. The horizontal axis represents PC 1 (explaining 30.43% of the total variance) and the vertical axis represents PC 2 (explaining 19.97% of the variance). Each colored symbol corresponds to a specific method: T-SQI (pink), aMA-SQI_phyl (light blue), aMA-SQI_cls (orange), aMA-SQI_ord (green), aMA-SQI_fam (purple), aMA-SQI_gen (dark blue), aMA-SQI_spp (coral), aBM-SQI_phyl (dark green), aBM-SQI_cls (yellow-orange), aMA-SQI_gen (red), aBM-SQI_gen (yellow), and a-SQI (olive green). The distribution of points reveals the relative similarity and divergence among the methods in the reduced two-dimensional PCA space.

**Figure 6:**
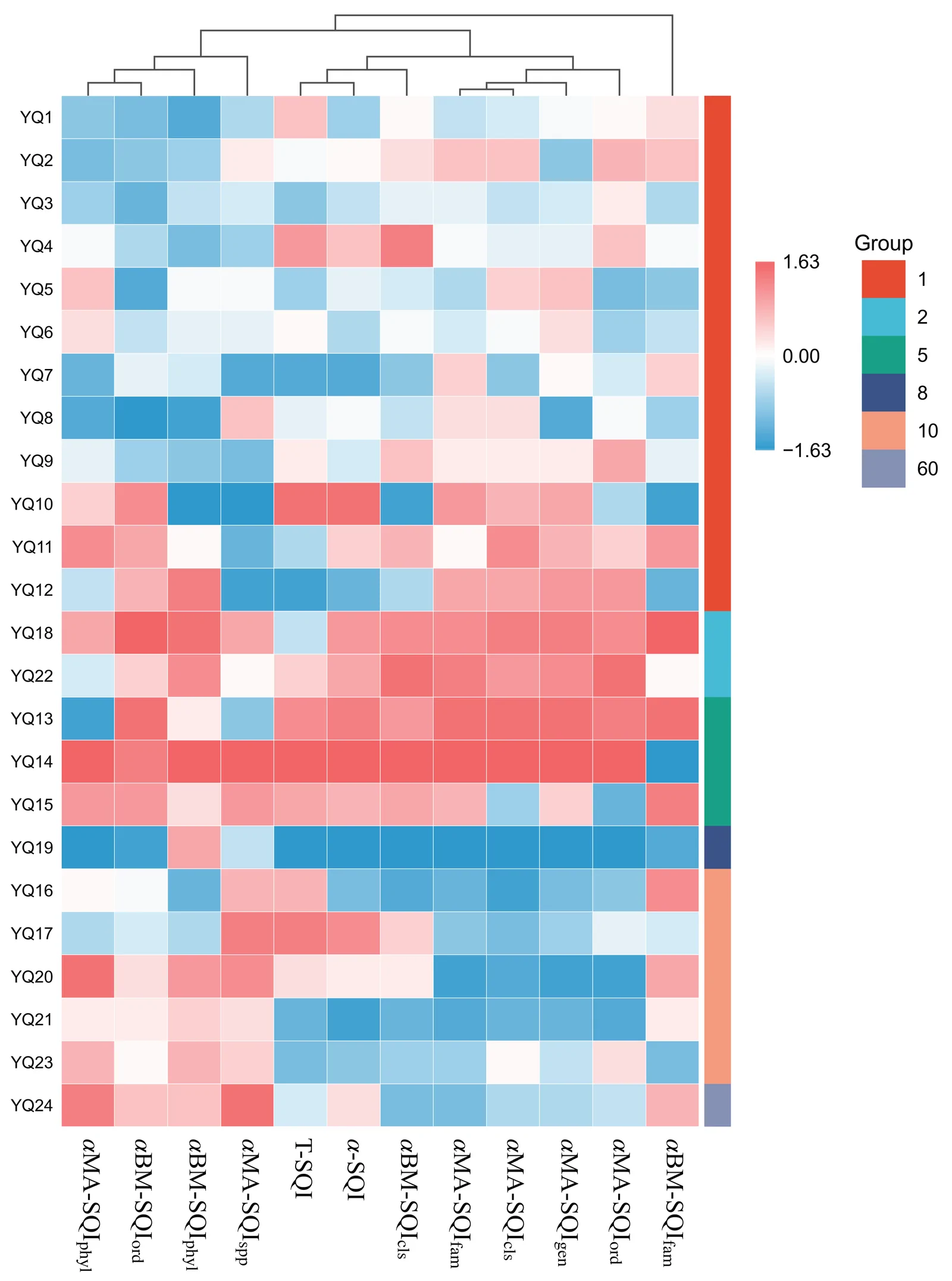
Variation in Rankings Across Different Sample by Different Calculation Methods. Heatmap with hierarchical dendrogram illustrating the variation in rankings across samples YQ1–YQ24 for different calculation methods (e.g., αMA-SQI_ply1, αBM-SQI_ord, T-SQI, α-SQI, etc.). Cell colors represent the ranking values on a scale from −1.63 (dark blue) to 1.63 (dark red), with the accompanying legend indicating the numeric range. The dendrogram above the heatmap clusters samples based on similarity of their ranking patterns, and the “Group” legend on the right assigns colors to predefined groups (1, 2, 5, 8, 10, 60).

The comparison between αMA-SQI and αBM-SQI further indicates that the indicator-selection strategy influenced SQI behavior. αMA-SQI was constructed using the most abundant taxa, whereas αBM-SQI emphasized taxa with stronger discriminatory characteristics. These approaches therefore capture different aspects of bacterial community structure and can produce different MDS compositions and ranking patterns. The PCA analysis in Figure 5 and the hierarchical clustering in Figure 6 further revealed distinct patterns in the ranking profiles generated by different SQI formulations across the sampling sites.

Importantly, the PCA and clustering results should be interpreted as differences in ranking profiles rather than as direct evidence of differences in microbial function. The observed separation among SQI formulations indicates that changes in the selected indicators propagated into the final classification of sampling sites. Among the tested formulations, αMA-SQIord and αMA-SQIfam showed comparatively stable ranking patterns. This suggests that intermediate taxonomic levels may provide a practical balance between the information retained from microbial community composition and the stability of soil-quality rankings in the present dataset. However, this observation is specific to the 24 site-level samples from one restored mining area and one sampling campaign and therefore requires independent validation.

### 4.4. Recurrent Microbial Indicators and Implications for SQI Construction

Several bacterial groups were repeatedly selected in the microbial-enhanced MDSs (Table 10). In particular, Gemmatimonadota and related taxonomic groups occurred in multiple αMA-SQI and αBM-SQI formulations. The repeated selection of this lineage suggests that variation in its relative abundance captured environmental information that was complementary to conventional physicochemical indicators. Gemmatimonadota are widely distributed in soils and are frequently associated with plant and rhizosphere environments [38], supporting their potential value as a taxonomic indicator of soil environmental conditions.

However, the ecological interpretation of these taxa should remain conservative. The present study used 16S rRNA gene amplicon sequencing to characterize bacterial community composition and relative abundance rather than directly measuring functional genes or process rates. Therefore, the repeated selection of Gemmatimonadota or other bacterial groups should be interpreted as evidence of their potential diagnostic value rather than as direct evidence of specific microbial functions. This distinction is important because taxonomic composition alone cannot establish changes in nitrogen cycling, pollutant degradation, or other ecosystem processes [34].

Overall, the present results support the incorporation of sequencing-derived microbial indicators as a complementary component of SQI construction in restored mining soils. Microbial integration altered site rankings and increased the biological representation of the SQI, while its effect depended on both indicator-selection strategy and taxonomic resolution. The relatively stable ranking patterns observed for αMA-SQIord and αMA-SQIfam identify these formulations as promising candidates for further evaluation, rather than universally optimal models. Future studies should validate the framework using independent restored mining sites, multiple sampling periods, and additional measurements of soil biological functions, including microbial biomass, enzyme activity, soil respiration, plant productivity, and functional-gene abundance.

## 5. Conclusions

This study established a novel evaluation framework for the Soil Quality Index (SQI) by integrating high-throughput sequencing data with traditional physicochemical parameters, demonstrating the critical importance of microbial indicators in soil quality assessment. Our findings reveal that incorporating α-diversity, gene abundance, and dominant species information significantly enhances the ecological rationality of SQI rankings, thereby improving the logical consistency between restoration duration and soil quality outcomes. The robust performance of long-term restoration sites (e.g., YQ19) across multiple models, coupled with the appropriately lower rankings of bare land sites (YQ14, YQ22), confirms the sensitivity of microbial community dynamics as indicators of ecological restoration progress. Gemmatimonadota emerged as a key functional taxon, consistently selected as a Minimum Data Set (MDS) component across multiple taxonomic levels, highlighting its diagnostic value for soil health assessment. Statistical analyses identified order- and family-level taxonomic units as exhibiting minimal ranking bias when utilized in MDS construction, recommending their adoption as core indicators in future SQI calculations. This research provides a more comprehensive biological perspective for soil quality evaluation and suggests that subsequent studies should further integrate microbial functional gene information, refine indicator selection methodologies, and validate their applicability across diverse ecological contexts.

## Figures and Tables

**Table 1:** Specific Details of the Sampling Point.

Sample	Ecosystem Type	Vegetation Type	Restoration Type	Restoration Years
YQ1	Plantation of Scots Pine	Scots Pine	Artificial	1
YQ2	Plantation of Scots Pine	Scots Pine	Artificial	1
YQ3	Plantation of Scots Pine	Scots Pine	Artificial	1
YQ4	Mixed Coniferous-Broadleaf Forest	Scots Pine-Elaeagnus	Artificial	1
YQ5	Mixed Coniferous-Broadleaf Forest	Scots Pine-Elaeagnus	Artificial	1
YQ6	Mixed Coniferous-Broadleaf Forest	Scots Pine-Elaeagnus	Artificial	1
YQ7	Plantation of Scots Pine	Scots Pine	Artificial	1
YQ8	Plantation of Scots Pine	Scots Pine	Artificial	1
YQ9	Plantation of Scots Pine	Scots Pine	Artificial	1
YQ10	Plantation of Scots Pine	Scots Pine	Artificial	1
YQ11	Plantation of Scots Pine	Scots Pine	Artificial	1
YQ12	Plantation of Scots Pine	Scots Pine	Artificial	1
YQ13	Plantation of Scots Pine	Scots Pine-Artemisia	Artificial	5
YQ14	Natural Soil	Scots Pine-Artemisia	Artificial	5
YQ15	Plantation of Scots Pine	Scots Pine-Armeniaca	Artificial	5
YQ16	Mixed Coniferous-Broadleaf Forest	Cherry-Scots Pine-Sabina-Cedrus	Artificial	10
YQ17	Plantation of Scots Pine	Scots Pine	Artificial	10
YQ18	Plantation of Scots Pine	Scots Pine	Artificial	2
YQ19	Plantation of Scots Pine	Scots Pine-Rosa acicularis	Artificial	8
YQ20	Secondary Forest of Black Locust	Black Locust-Sorbaria-Schrebera	Natural	10
YQ21	Secondary Forest of Scots Pine	Scots Pine	Natural	10
YQ22	Natural Grassland	Artemisia-China Toon	Natural	2
YQ23	Mixed Coniferous-Broadleaf Forest	Scots Pine-Prunus mume	Artificial	10
YQ24	Secondary Forest of Scots Pine	Scots Pine-Vitex negundo	Natural	60

**Table 2:** Microbial sequencing parameters.

Sequencing Primers	Sequencing Region	Database	Main Methods
F: ACTCCTACGGGAGGCAGCA R: GGACTACHVGGGTWTCTAAT	16S_V3V4	SILVA 138	DADA2

**Table 3:** List of Abbreviations. The table presents the abbreviations used in the study together with their full definitions. It is arranged in four columns: two pairs of “Abbreviation” and “Full Name,” allowing more entries to be displayed compactly. Key entries include: T-SQI—Traditional Soil Quality Index based only on physicochemical properties. α-SQI—Soil Quality Index that adds bacterial α-diversity to the physicochemical data. αMA-SQI and αBM-SQI—Indices that further incorporate the relative abundances of the five most abundant bacterial taxa. Each of these has sub-variants indicating the taxonomic level (phylum, class, order, family, genus, species) at which the abundances are considered.

Abbreviation	Full Name	Abbreviation	Full Name
T-SQI	Traditional SQI focusing solely on soil physicochemical properties		
α-SQI	Incorporation of soil physicochemical properties and α-diversity of bacterial communities into the SQI		
αMA-SQI	Incorporation of soil physicochemical properties, α-diversity of bacterial communities and relative abundances of the five most abundant bacteria into the SQI	αMA-SQI_phyl_	relative abundances of the five most abundant bacteria at phylum
		αMA-SQI_cls_	relative abundances of the five most abundant bacteria at class
		αMA-SQI_ord_	relative abundances of the five most abundant bacteria at order
		αMA-SQI_fam_	relative abundances of the five most abundant bacteria at family
		αMA-SQI_gen_	relative abundances of the five most abundant bacteria at genus
		αMA-SQI_spp_	relative abundances of the five most abundant bacteria at species
αBM-SQI	Incorporation of soil physicochemical properties, α-diversity of bacterial communities and relative abundances of the five most abundant bacteria into the SQI	αBM-SQI_phyl_	relative abundances of the five most abundant bacteria at phylum
		αBM-SQI_cls_	relative abundances of the five most abundant bacteria at class
		αBM-SQI_ord_	relative abundances of the five most abundant bacteria at order
		αBM-SQI_fam_	relative abundances of the five most abundant bacteria at family

**Table 4:** Analysis of Soil Microbial Species Composition in Different Plots. Summary of soil microbial taxonomic unit numbers across samples YQ1–YQ24. Rows represent individual soil samples; columns represent successive microbial taxonomic ranks (domain, phylum, class, order, family, genus, species). Values indicate the number of taxonomic units identified for each rank per sample.

Sample	Domain	Phylum	Class	Order	Family	Genus	Species
YQ1	1	5.67	10.67	26.33	40.67	65	80.33
YQ2	1	7.67	13	34	54.67	88	106.33
YQ3	1	5.33	11.67	26.67	39	62.33	75.33
YQ4	1	5.67	12.67	26.33	40	55	63
YQ5	1	4.67	10.67	29	58.67	92	113.33
YQ6	1	5.67	12.67	31	55.33	89.67	119.67
YQ7	1	6	12	27.33	44.67	60.67	72.67
YQ8	1	7.33	12.67	34.67	54.67	87.33	107
YQ9	1	5	11.67	30.67	44.33	69.33	87.67
YQ10	1	3.67	11.67	29.67	45.67	71.33	84.67
YQ11	1	7	12.67	34.33	56.33	94.67	109
YQ12	1	4.33	10.33	20.33	35.33	46.33	53
YQ13	1	4	12	33.33	60.67	92.33	110
YQ14	1	4	8.67	17.33	25.33	24.33	25.33
YQ15	1	3.33	10	28.33	45.33	61	69.33
YQ16	1	8.67	17	44.33	72.33	122	150.33
YQ17	1	4.33	9.67	26	42.67	60.67	73.67
YQ18	1	2.67	9.33	26	51	67	78.33
YQ19	1	6	14.33	44	79	110.33	149.33
YQ20	1	5.67	11	40	78.33	118	153
YQ21	1	4	9.33	26.67	50.67	68	87.67
YQ22	1	5.67	13	29.67	50.67	66.33	78
YQ23	1	5	11.33	35.33	68.67	111.33	144
YQ24	1	4	12	32	51	76.67	96

**Table 5:** Principal Component Analysis Results. Summary of principal component analysis for soil physicochemical variables. Columns represent the first three principal components (PC-1, PC-2, PC-3). Rows list eigenvalues, variance-explained proportions, cumulative variance proportions, and eigenvector loadings of each soil property. Values indicate calculated PCA results.

PCs	PC-1	PC-2	PC-3
Eigenvalue	3.635	2.056	1.206
Percentage of variance explained (%)	40.393	22.850	13.398
Cumulative percentage (%)	40.393	63.243	76.641
Eigenvector			
EC	0.889	0.260	−0.034
AK	0.871	−0.104	0.236
Na	0.837	0.245	−0.229
TP	0.773	−0.015	0.365
TN	−0.529	0.488	0.110
pH	−0.013	0.828	0.293
SOM	0.470	−0.797	−0.125
AP	0.530	0.540	−0.117
AN	−0.090	−0.260	0.914

**Table 6:** Different Calculation Methods of MDS. Lists a series of MDS (Multidimensional Scaling) calculation methods in the first column and the corresponding variables used for each method in the second column.

	MDS
T-SQI	EC, pH, AN
α-SQI	EC, pH, AN, Observed_species
αMA-SQI_phyl_	AN, EC, Faith_pd, Chloroflexi, p__Proteobacteria, Gemmatimonadaceae
αMA-SQI_cls_	AN, Gemmatimonadetes, c__Vicinamibacteria, c__Blastocatellia, c__Alphaproteobacteria
αMA-SQI_ord_	AN, AK, Burkholderiales, Vicinamibacterales, o__Pyrinomonadales, Gemmatimonadales
αMA-SQI_fam_	EC, AN, Vicinamibacteraceae, Gemmatimonadaceae, Nocardioidaceae, f__Pyrinomonadaceae
αMA-SQI_gen_	AN, species, Vicinamibacteraceae, g__RB41, g__Allorhizobium-Neorhizobium-Pararhizobium-Rhizobium
αMA-SQI_spp_	AN, Chao1, Archangium_gephyra, Sporichthya_polymorpha
αBM-SQI_phyl_	EC, species, Observed_species, Proteobacteria, Gemmatimonadota
αBM-SQI_cls_	EC, AN, Observed_species, Alphaproteobacteria
αBM-SQI_ord_	AN, Na, Chao1, Gemmatimonadales
αBM-SQI_fam_	AN, TN, Na, Faith_pd, Gemmatimonadaceae, Sphingomonadaceae, 67-14

**Table 7:** Ranking of SQI for Different Taxonomic Units Across Various Sampling Samples. The table presents the ranking results of the SQI (Soil Quality Index) for various taxonomic groups across 24 sampling sites (YQ1–YQ24). Each row corresponds to a sample, and the columns list the rank positions of different SQI metrics (e.g., T-SQ, αMA-SQI, αBM-SQI, α-SQ) for that sample. The numbers indicate how each metric ranks within the respective sample.

Sample	T-SQI	αMA-SQI_phyl_	αMA-SQI_cls_	αMA-SQI_ord_	αMA-SQI_fam_	αMA-SQI_gen_	αMA-SQI_spp_	αBM-SQI_phyl_	αBM-SQI_cls_	αBM-SQI_ord_	αBM-SQI_fam_	α-SQI
YQ1	17	6	10	12	9	13	8	3	13	5	15	7
YQ2	12	5	17	6	17	18	14	7	15	6	17	13
YQ3	6	7	9	10	11	14	10	9	11	4	8	9
YQ4	20	12	11	11	12	17	7	5	22	8	12	17
YQ5	7	17	16	17	8	5	12	12	10	3	6	11
YQ6	13	15	12	15	10	7	11	11	12	9	9	8
YQ7	3	4	6	13	16	10	3	10	6	11	16	3
YQ8	11	3	15	3	15	12	17	2	9	1	7	12
YQ9	14	11	14	14	14	19	5	6	17	7	11	10
YQ10	23	16	18	19	20	8	1	1	2	21	2	23
YQ11	8	21	21	18	13	16	4	13	18	19	20	16
YQ12	2	9	19	20	19	20	2	22	8	18	4	4
YQ13	21	2	23	23	23	22	6	14	20	23	23	22
YQ14	24	24	24	24	24	24	24	24	24	22	1	24
YQ15	19	20	7	16	18	4	20	15	19	20	22	18
YQ16	18	13	2	5	4	6	18	4	3	12	21	5
YQ17	22	8	5	7	6	11	22	8	16	10	10	21
YQ18	9	19	22	22	21	21	19	23	21	24	24	20
YQ19	1	1	1	1	1	1	9	19	1	2	3	1
YQ20	15	23	3	2	2	2	21	20	14	15	19	14
YQ21	4	14	4	4	3	3	15	16	4	14	14	2
YQ22	16	10	20	21	22	23	13	21	23	16	13	19
YQ23	5	18	13	9	7	15	16	18	7	13	5	6
YQ24	10	22	8	8	5	9	23	17	5	17	18	15

**Table 8:** SQI Calculation Results for Different Taxonomic Units. The table shows the Soil Quality Index (SQI) results for a series of samples labeled YQ1 through YQ24. Each row corresponds to one sample, and the columns present different SQI metrics—such as T-SQI, aMA-SQI (with sub-categories p, c, o, f, g, s), αBM-SQI (with sub-categories p, c, o), and a-SQI. The cells contain the numerical values obtained for each metric in each sample.

Sample	T-SQI	αMA-SQI_P_	αMA-SQI_C_	αMA-SQI_O_	αMA-SQI_F_	αMA-SQI_G_	αMA-SQI_S_	αBM-SQI_P_	αBM-SQI_C_	αBM-SQI_O_	αBM-SQI_F_	α-SQI
YQ1	0.70	1.92	2.25	2.35	1.68	2.01	2.27	1.60	1.16	1.71	1.94	0.80
YQ2	0.71	2.02	1.84	3.42	1.47	1.88	1.44	1.51	1.15	1.70	1.89	0.76
YQ3	0.83	1.90	2.30	2.40	1.66	1.95	2.00	1.50	1.17	1.71	2.18	0.79
YQ4	0.57	1.81	2.21	2.38	1.66	1.89	2.65	1.54	1.00	1.63	2.02	0.69
YQ5	0.79	1.70	1.86	2.04	1.71	2.85	1.91	1.38	1.18	1.76	2.28	0.77
YQ6	0.71	1.72	2.10	2.31	1.68	2.56	1.92	1.41	1.17	1.62	2.18	0.79
YQ7	1.15	2.10	2.34	2.32	1.53	2.22	3.57	1.41	1.30	1.49	1.94	0.88
YQ8	0.75	2.14	1.91	4.30	1.56	2.07	1.29	1.61	1.20	2.09	2.26	0.77
YQ9	0.71	1.81	2.00	2.32	1.63	1.72	3.21	1.51	1.13	1.68	2.03	0.77
YQ10	0.47	1.72	1.83	1.62	1.24	2.43	4.51	1.63	1.46	1.21	2.66	0.57
YQ11	0.79	1.62	1.46	2.01	1.66	1.91	3.24	1.36	1.11	1.30	1.78	0.74
YQ12	1.26	1.83	1.64	1.48	1.25	1.42	3.58	1.04	1.29	1.33	2.38	0.84
YQ13	0.57	2.25	1.29	1.07	0.79	1.05	3.16	1.31	1.09	1.11	1.60	0.60
YQ14	0.29	1.20	0.94	0.78	0.58	0.60	0.98	0.74	0.64	1.18	3.16	0.38
YQ15	0.64	1.67	2.31	2.27	1.45	3.18	1.21	1.28	1.10	1.26	1.68	0.68
YQ16	0.67	1.75	3.11	3.53	2.13	2.76	1.24	1.59	1.45	1.48	1.74	0.84
YQ17	0.50	1.85	2.53	3.10	1.88	2.13	1.11	1.51	1.15	1.51	2.07	0.60
YQ18	0.79	1.67	1.34	1.33	1.12	1.15	1.24	1.01	1.03	1.10	1.56	0.62
YQ19	2.31	2.28	4.85	6.29	4.39	5.33	2.20	1.18	1.95	2.04	2.59	1.47
YQ20	0.71	1.51	3.02	4.35	2.93	3.30	1.17	1.18	1.16	1.37	1.87	0.76
YQ21	1.05	1.72	2.75	3.88	2.51	3.20	1.41	1.25	1.41	1.39	1.97	0.90
YQ22	0.71	1.82	1.55	1.42	1.09	0.98	1.56	1.11	0.98	1.36	2.01	0.63
YQ23	0.91	1.69	2.05	2.63	1.78	1.92	1.31	1.23	1.29	1.47	2.31	0.82
YQ24	0.79	1.57	2.31	2.86	1.93	2.27	1.07	1.24	1.39	1.34	1.88	0.75

**Table 9:** The correlation between SQI and microbial α-diversity, as well as its significance. The table presents the correlation analysis between several soil-quality indices (SQIs) and microbial alpha-diversity metrics. Rows list the alpha-diversity indices (e.g., Chao1, Shannon, Simpson, Faith’s PD). Columns show different SQI categories (such as T-SQI, αBM-SQI_phyl, αBM-SQI_cls, α-SQI, αMA-SQI_phyl, αMA-SQI_cls). For each SQI-diversity pair, the table provides the correlation coefficient and the one-tailed significance value, indicating the strength and statistical significance of the relationship.

	α Diversity	T-SQI	αBM-SQI_phyl_	αBM-SQI_cls_	αBM-SQI_ord_	αBM-SQI_fam_	α-SQI
Correlation	Chao1	0.225	0.703	0.485	0.680	−0.230	0.577
	Faith_pd	0.192	0.658	0.480	0.643	−0.275	0.534
	Goods_coverage	−0.007	−0.553	−0.036	−0.478	0.208	−0.226
	Observed_species	0.238	0.687	0.512	0.674	−0.223	0.590
	Pielou_e	0.305	0.641	0.485	0.606	−0.342	0.594
	Shannon	0.279	0.686	0.511	0.648	−0.304	0.603
	Simpson	0.338	0.428	0.328	0.570	−0.444	0.571
Significance (One-Tailed)	Chao1	0.146	0.000	0.008	0.000	0.139	0.002
	Faith_pd	0.184	0.000	0.009	0.000	0.097	0.004
	Goods_coverage	0.487	0.003	0.434	0.009	0.165	0.144
	Observed_species	0.131	0.000	0.005	0.000	0.147	0.001
	Pielou_e	0.073	0.000	0.008	0.001	0.051	0.001
	Shannon	0.093	0.000	0.005	0.000	0.074	0.001
	Simpson	0.053	0.019	0.059	0.002	0.015	0.002
	α Diversity	αMA-SQI_phyl_	αMA-SQI_cls_	αMA-SQI_ord_	αMA-SQI_fam_	αMA-SQI_gen_	αMA-SQI_spp_
Correlation	Chao1	0.372	0.634	0.652	0.591	0.614	−0.050
	Faith_pd	0.331	0.565	0.717	0.579	0.574	−0.162
	Goods_coverage	−0.437	−0.255	−0.137	−0.140	−0.239	−0.106
	Observed_species	0.351	0.646	0.681	0.615	0.628	−0.068
	Pielou_e	0.408	0.543	0.549	0.516	0.506	0.056
	Shannon	0.398	0.598	0.619	0.569	0.573	0.010
	Simpson	0.430	0.456	0.490	0.450	0.389	−0.104
Significance (One-Tailed)	Chao1	0.037	0.000	0.000	0.001	0.001	0.408
	Faith_pd	0.057	0.002	0.000	0.002	0.002	0.225
	Goods_coverage	0.016	0.115	0.261	0.257	0.130	0.312
	Observed_species	0.046	0.000	0.000	0.001	0.001	0.376
	Pielou_e	0.024	0.003	0.003	0.005	0.006	0.398
	Shannon	0.027	0.001	0.001	0.002	0.002	0.481
	Simpson	0.018	0.013	0.007	0.014	0.030	0.315

**Table 10:** Comparison of SQI Calculation for Species and Dominant Species. The table lists taxonomic groups (phyla, classes, orders, families) alongside several SQI metrics labeled αMA-SQI and αBM-SQI with subscripts indicating the taxonomic level (e.g., phy, cls, ord, fam). Each column shows which taxa are considered dominant or relevant for that particular SQI calculation, providing a side-by-side overview of species-level and higher-level groupings used in the SQI analysis.

αMA-SQI_phyl_	αBM-SQI_phyl_	αMA-SQI_cls_	αBM-SQI_cls_
Acidobacteriota	Actinobacteriota	Thermoleophilia	Actinobacteria
Actinobacteriota	Proteobacteria	Gammaproteobacteria	Thermoleophilia
Chloroflexi	Acidobacteriota	Actinobacteria	Alphaproteobacteria
Proteobacteria	Gemmatimonadota	Gemmatimonadetes	Gammaproteobacteria
Gemmatimonadota	Chloroflexi	Alphaproteobacteria	Acidimicrobiia
αMA-SQI_ord_	αBM-SQI_ord_	αMA-SQI_fam_	αBM-SQI_fam_
Gemmatimonadales	Gemmatimonadales	Vicinamibacteraceae	Gemmatimonadaceae
Solirubrobacterales	Solirubrobacterales	Solirubrobacteraceae	Vicinamibacteraceae
Vicinamibacterales	Vicinamibacterales	Nocardioidaceae	Solirubrobacteraceae
Burkholderiales	Burkholderiales	Gemmatimonadaceae	Sphingomonadaceae
Sphingomonadales	Sphingomonadales	Sphingomonadaceae	67-14

## Data Availability

The original contributions presented in this study are included in the article/Supplementary Materials. Further inquiries can be directed to the corresponding author.

## Supplementary Materials

The following supporting information can be downloaded at https://www.mdpi.com/article/10.3390/microorganisms14092009/s1.
