# Supplementary material for: Integrating Microbial Indicators from High-Throughput Sequencing into Soil Quality Index: A Case Study in a Restored Mining Area
Source: Microorganisms. 2026 Sep 10;14(9):2009. doi: 10.3390/microorganisms14092009 (PMC13609458; doi:10.3390/microorganisms14092009)
Supplement: Supplementary file 1 [file microorganisms-14-02009-s001.zip › microorganisms-4493622-supplementary.pdf]

Table S1 Specific Details of the Sampling Point

Sample	Ecosystem Type	Vegetation Type	Restoration Type	Restoration Years
YQ1	Plantation of Scots Pine	Scots Pine	Artificial	1
YQ2	Plantation of Scots Pine	Scots Pine	Artificial	1
YQ3	Plantation of Scots Pine	Scots Pine	Artificial	1
YQ4	Mixed Coniferous-Broadleaf Forest	Scots Pine-Elaeagnus	Artificial	1
YQ5	Mixed Coniferous-Broadleaf Forest	Scots Pine-Elaeagnus	Artificial	1
YQ6	Mixed Coniferous-Broadleaf Forest	Scots Pine-Elaeagnus	Artificial	1
YQ7	Plantation of Scots Pine	Scots Pine	Artificial	1
YQ8	Plantation of Scots Pine	Scots Pine	Artificial	1
YQ9	Plantation of Scots Pine	Scots Pine	Artificial	1
YQ10	Plantation of Scots Pine	Scots Pine	Artificial	1
YQ11	Plantation of Scots Pine	Scots Pine	Artificial	1
YQ12	Plantation of Scots Pine	Scots Pine	Artificial	1
YQ13	Plantation of Scots Pine	Scots Pine- Artemisia	Artificial	5
YQ14	Natural Soil	Scots Pine- Artemisia	Artificial	5
YQ15	Plantation of Scots Pine	Scots Pine- Armeniaca	Artificial	5
YQ16	Mixed Coniferous-Broadleaf Forest	Cherry-Scots Pine-Sabina-Cedrus	Artificial	10
YQ17	Plantation of Scots Pine	Scots Pine	Artificial	10
YQ18	Plantation of Scots Pine	Scots Pine	Artificial	2
YQ19	Plantation of Scots Pine	Scots Pine- Rosa acicularis	Artificial	8
YQ20	Secondary Forest of Black Locust	Black Locust-Sorbaria-Schrebera	Natural	10
YQ21	Secondary Forest of Scots Pine	Scots Pine	Natural	10
YQ22	Natural Grassland	Artemisia - China Toon	Natural	2
YQ23	Mixed Coniferous-Broadleaf Forest	Scots Pine-Prunus mume	Artificial	10
YQ24	Secondary Forest of Scots Pine	Scots Pine- Vitex negundo	Natural	60

Table S2 Microbial sequencing parameters

Sequencing Primers	Sequencing Region	Database	Main Methods
F:ACTCCTACGGGAGGCAGCA<br>R:CGGACTACHVGGGTWTCTAAT	16S_V3V4	silva_132	dada2

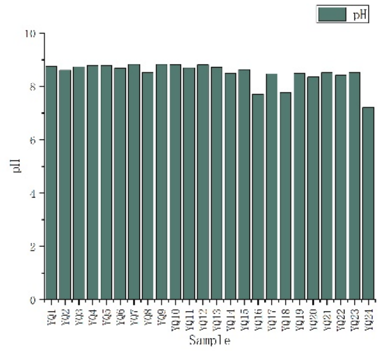

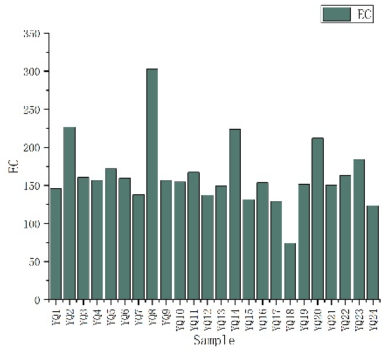

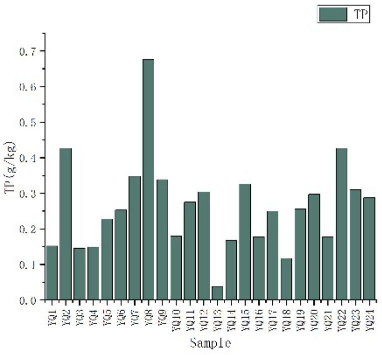

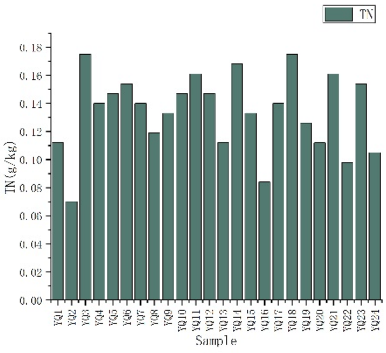

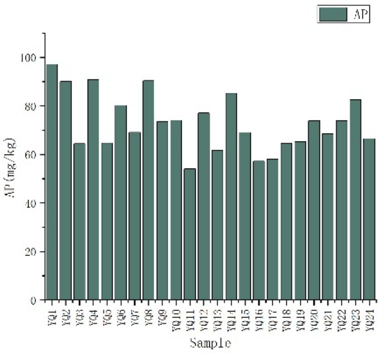

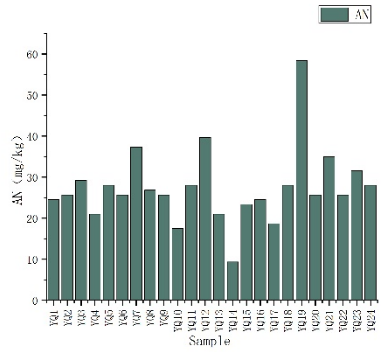

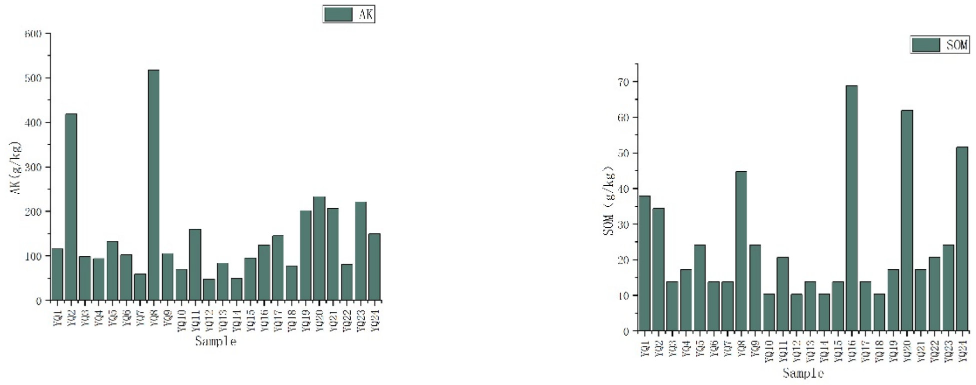

Fig.S1 Soil physicochemical properties under different samples

Table S3 Total Variance Explained (T-SQI)

	Initial Eigenvalue			Sum of Squared Loadings		
PC	Total	Percentage of Variance	Cumulative Percentage	Total	Percentage of Variance	Cumulative Percentage
1	3.635	40.393	40.393	3.635	40.393	40.393
2	2.056	22.850	63.243	2.056	22.850	63.243
3	1.206	13.398	76.641	1.206	13.398	76.641
4	.718	7.977	84.618			
5	.497	5.524	90.142			
6	.379	4.209	94.352			
7	.228	2.539	96.891			
8	.201	2.233	99.124			
9	.079	.876	100.000			

Table S4 Principal Component Analysis Results (T-SQI)

PCs	PC-1	PC-2	PC-3
Eigenvalue	3.635	2.056	1.206
Variance Explanation Percentage（%）	40.393	22.850	13.398
Cumulative percentage（%）	40.393	63.243	76.641
Feature vector			
EC	.889	.260	-.034
AK	.871	-.104	.236
Na	.837	.245	-.229
TP	.773	-.015	.365
TN	-.529	.488	.110
pH	-.013	.828	.293
SOM	.470	-.797	-.125
AP	.530	.540	-.117
AN	-.090	-.260	.914

Table S5 Correlation Matrix (T-SQI)

		pH	EC	SOM	AN	TN	AP	TP	Na	AK
Correlation	pH	1.000								
	EC	.233	1.000							
	SOM	-.630	.284	1.000						
	AN	.010	-.178	.060	1.000					
	TN	.271	-.252	-.577	.012	1.000				
	AP	.291	.500	-.189	-.193	-.170	1.000			
	TP	.045	.612	.283	.166	-.347	.311	1.000		
	Na	.075	.820	.219	-.301	-.226	.490	.521	1.000	
	AK	-.062	.734	.433	.142	-.397	.329	.678	.649	1.000

Table S6 Total Variance Explained (αMA-SQI_{phyl})

PC	Initial Eigenvalue			Sum of Squared Loadings		
	Total	Percentage of Variance	Cumulative Percentage	Total	Percentage of Variance	Cumulative Percentage
1	6.827	42.666	42.666	6.827	42.666	42.666
2	3.250	20.312	62.979	3.250	20.312	62.979
3	2.004	12.526	75.504	2.004	12.526	75.504
4	1.355	8.467	83.971	1.355	8.467	83.971
5	.717	4.484	88.456			
6	.557	3.479	91.935			
7	.397	2.478	94.413			
8	.310	1.938	96.352			
9	.224	1.401	97.753			
10	.161	1.006	98.759			
11	.112	.698	99.458			
12	.060	.377	99.835			
13	.020	.127	99.963			
14	.006	.035	99.998			
15	.000	.002	100.000			
16	5.907E-5	.000	100.000			

Table S7 Principal Component Analysis Results (αMA-SQI_{phyl})

PCs	PC-1	PC-2	PC-3	PC-4
Eigenvalue	6.827	3.250	2.004	1.355
Variance Explanation Percentage（%）	42.666	20.312	12.526	8.467
Cumulative percentage（%）	42.666	62.979	75.504	83.971
Feature vector				
Observed_species	.947	-.216	.053	-.126
Faith_pd	.946	-.036	-.066	-.088

Shannon	.944	-.286	.040	.038
Chao1	.936	-.242	.109	-.156
Pielou_e	.897	-.323	.024	.149
Simpson	.828	-.303	-.005	.212
SOM	.627	.255	-.583	-.325
TN	-.545	-.251	.372	.286
Na	.239	.854	.175	-.082
EC	.387	.820	.204	.079
AK	.611	.629	-.072	.227
TP	.408	.618	-.060	.507
A	.187	.538	.501	-.204
pH	.044	-.042	.899	.196
Goods_coverage	-.453	.351	-.596	.403
AN	.312	-.378	-.102	.698

Table S8 Total Variance Explained (αMA-SQI_{cls})

PC	Initial Eigenvalue			Sum of Squared Loadings		
	Total	Percentage of Variance	Cumulative Percentage	Total	Percentage of Variance	Cumulative Percentage
1	8.348	49.104	49.104	8.348	49.104	49.104
2	2.539	14.937	64.041	2.539	14.937	64.041
3	1.748	10.283	74.324	1.748	10.283	74.324
4	1.465	8.618	82.943	1.465	8.618	82.943
5	1.217	7.161	90.104	1.217	7.161	90.104
6	.616	3.626	93.730			
7	.418	2.457	96.187			
8	.243	1.429	97.616			
9	.189	1.113	98.729			
10	.120	.706	99.435			
11	.072	.423	99.858			
12	.011	.064	99.922			
13	.008	.049	99.971			
14	.003	.016	99.988			
15	.001	.008	99.995			
16	.001	.004	99.999			
17	.000	.001	100.000			

Table S9 Principal Component Analysis Results (αMA-SQI_{cls})

PCs	PC-1	PC-2	PC-3	PC-4	PC-5
Eigenvalue	8.348	2.539	1.748	1.465	1.217
Variance Explanation Percentage （%）	49.104	14.937	10.283	8.618	7.161
Cumulative percentage（%）	49.104	64.041	74.324	82.943	90.104

Feature vector					
Faith_pd	.938	.228	-.078	.041	.032
Acidobacteriota	.928	.030	-.177	-.207	-.147
p__Actinobacteriota	-.922	-.038	-.247	-.042	-.115
Chao1	.920	.058	-.146	.251	.077
Shannon	.917	.040	-.171	.248	.196
p__Acidobacteriota	.890	-.100	-.092	-.090	-.122
Pielou_e	.858	.015	-.185	.264	.282
Actinobacteriota	-.856	-.035	-.461	-.067	-.084
p__Chloroflexi	-.641	.494	.347	.067	.348
SOM	.626	.316	-.050	-.186	-.352
EC	.247	.759	.121	-.217	-.285
Chloroflexi	-.403	.707	.349	-.021	.415
AK	.526	.647	.176	-.328	.007
p__Proteobacteria	.258	-.607	.706	-.063	.102
Proteobacteria	.589	-.446	.593	-.216	-.010
Gemmatimonadota	.082	.202	.138	.919	-.086
AN	.292	-.149	-.429	-.349	.720

Table S10 Total Variance Explained (αMA-SQI_{ord})

	Initial Eigenvalue			Sum of Squared Loadings		
PC	Total	Percentage of Variance	Cumulative Percentage	Total	Percentage of Variance	Cumulative Percentage
1	5.943	39.622	39.622	5.943	39.622	39.622
2	2.586	17.238	56.860	2.586	17.238	56.860
3	1.649	10.995	67.855	1.649	10.995	67.855
4	1.348	8.985	76.840	1.348	8.985	76.840
5	1.210	8.068	84.909	1.210	8.068	84.909
6	.861	5.737	90.646			
7	.469	3.124	93.770			
8	.401	2.671	96.440			
9	.208	1.383	97.824			
10	.167	1.115	98.939			
11	.063	.417	99.355			
12	.039	.261	99.616			
13	.028	.189	99.805			
14	.020	.130	99.935			
15	.010	.065	100.000			

Table S11 Principal Component Analysis Results (αMA-SQI_{ord})

PCs	PC-1	PC-2	PC-3	PC-4	PC-5
Eigenvalue	5.943	2.586	1.649	1.348	1.21
Variance Explanation Percentage	39.622	17.238	10.995	8.985	8.068

(%)					
Cumulative percentage (%)	39.622	56.86	67.855	76.84	84.909
Feature vector					
c__Vicinamibacteria	.928	.120	.071	.031	.087
Chao1	.878	-.238	.268	.087	.127
Thermoleophilia	-.871	-.209	.269	.098	.135
Shannon	.866	-.220	.295	.131	.047
Gammaproteobacteria	.819	.377	-.349	.144	-.002
c__Gammaproteobacteria	.679	.557	-.300	.259	.093
species	.627	.509	.077	-.274	-.352
Actinobacteria	-.613	.207	.173	.139	-.291
AK	.534	-.299	-.065	-.369	-.447
c__Blastocatellia	.350	-.752	-.117	.437	.217
pH	.118	.627	.567	.148	-.077
SOM	.515	-.583	-.273	-.397	.064
c__Alphaproteobacteria	-.254	.413	-.638	.260	.322
AN	.216	-.300	.127	.739	-.486
Gemmatimonadetes	.324	.141	.572	-.102	.593

Table S12 Total Variance Explained (αMA-SQI_{fam})

	Initial Eigenvalue			Sum of Squared Loadings		
PC	Total	Percentage of Variance	Cumulative Percentage	Total	Percentage of Variance	Cumulative Percentage
1	7.724	51.496	51.496	7.724	51.496	51.496
2	2.063	13.756	65.252	2.063	13.756	65.252
3	1.384	9.226	74.477	1.384	9.226	74.477
4	1.227	8.179	82.656	1.227	8.179	82.656
5	1.014	6.758	89.414	1.014	6.758	89.414
6	.517	3.449	92.864			
7	.427	2.850	95.713			
8	.233	1.556	97.269			
9	.232	1.544	98.813			
10	.082	.548	99.360			
11	.042	.283	99.643			
12	.027	.179	99.822			
13	.017	.113	99.935			
14	.009	.060	99.995			

Table S13 Principal Component Analysis Results (αMA-SQI_{fam})

PCs	PC-1	PC-2	PC-3	PC-4	PC-5
Eigenvalue	7.724	2.063	1.384	1.227	1.014
Variance Explanation Percentage (%)	51.496	13.756	9.226	8.179	6.758

Cumulative percentage（%）	51.496	65.252	74.477	82.656	89.414
Feature vector					
Vicinamibacterales	.938	-.090	-.163	-.011	-.124
Shannon	.933	.197	.179	.163	.098
Faith_pd	.910	.194	-.195	.141	.023
Pielou_e	.899	.185	.259	.144	.184
o__Vicinamibacterales	.882	-.250	.050	-.065	-.182
Solirubrobacterales	-.856	.323	.085	.170	.056
Simpson	.779	.276	.266	.169	.321
o__Xanthomonadales	.752	-.461	.025	-.308	-.137
Burkholderiales	.623	-.055	.237	-.469	-.456
o__Pyrinomonadales	.358	.835	-.004	-.093	-.340
species	.608	-.635	-.073	.002	.390
AK	.516	.089	-.638	.045	.218
SOM	.542	.273	-.553	.304	-.214
Gemmatimonadales	.335	-.149	.558	.620	-.189
AN	.310	.530	.234	-.544	.438

Table S14 Total Variance Explained (αMA-SQI$_{gen}$)

	Initial Eigenvalue			Sum of Squared Loadings		
PC	Total	Percentage of Variance	Cumulative Percentage	Total	Percentage of Variance	Cumulative Percentage
1	8.911	46.900	46.900	8.911	46.900	46.900
2	2.453	12.909	59.809	2.453	12.909	59.809
3	2.210	11.632	71.441	2.210	11.632	71.441
4	1.492	7.854	79.295	1.492	7.854	79.295
5	.978	5.145	84.440			
6	.781	4.110	88.550			
7	.626	3.293	91.842			
8	.448	2.355	94.198			
9	.322	1.696	95.893			
10	.261	1.373	97.266			
11	.220	1.156	98.421			
12	.150	.791	99.212			
13	.066	.345	99.557			
14	.046	.241	99.798			
15	.018	.096	99.894			
16	.016	.082	99.976			
17	.003	.017	99.993			
18	.001	.006	99.999			
19	.000	.001	100.000			

Table S15 Principal Component Analysis Results (αMA-SQI_{gen})

PCs	PC-1	PC-2	PC-3	PC-4
Eigenvalue	8.911	2.453	2.21	1.492
Variance Explanation Percentage（%）	46.9	12.909	11.632	7.854
Cumulative percentage（%）	46.9	59.809	71.441	79.295
Feature vector				
Vicinamibacteraceae	.940	.110	-.126	.091
Chao1	.939	-.083	.212	-.134
Shannon	.938	-.116	.260	-.003
Faith_pd	.935	.148	.096	.013
f__Vicinamibacteraceae	.897	-.101	-.156	-.001
Pielou_e	.889	-.163	.286	.060
Solirubrobacteraceae	-.851	.160	.313	-.171
f__Xanthomonadaceae	.802	.095	-.416	.127
Simpson	.775	-.108	.376	.037
species	.593	-.336	-.547	.262
SOM	.553	.484	.035	-.091
EC	.337	.620	-.483	-.127
f__Rhizobiaceae	.223	-.590	-.260	.157
AK	.562	.582	-.279	.148
Goods_coverage	-.504	.520	-.038	.432
f__Pyrinomonadaceae	.362	.357	.726	-.027
Gemmatimonadaceae	.369	-.487	.058	-.620
Nocardioidaceae	-.309	-.511	.013	.598
AN	.277	.020	.586	.589

Table S16 Total Variance Explained (αMA-SQI_{spp})

	Initial Eigenvalue			Sum of Squared Loadings		
PC	Total	Percentage of Variance	Cumulative Percentage	Total	Percentage of Variance	Cumulative Percentage
1	8.096	50.599	50.599	8.096	50.599	50.599
2	2.152	13.453	64.051	2.152	13.453	64.051
3	1.815	11.344	75.396	1.815	11.344	75.396
4	1.132	7.076	82.472	1.132	7.076	82.472
5	.766	4.788	87.260			
6	.702	4.390	91.650			
7	.462	2.888	94.538			
8	.385	2.403	96.941			
9	.170	1.065	98.006			
10	.121	.759	98.765			
11	.089	.556	99.321			
12	.053	.330	99.651			

13	.036	.224	99.875
14	.017	.104	99.979
15	.003	.020	99.999
16	.000	.001	100.000

Table S17 Principal Component Analysis Results (αMA-SQI$_{spp}$)

PCs	PC-1	PC-2	PC-3	PC-4
Eigenvalue	8.096	2.152	1.815	1.132
Variance Explanation Percentage（%）	50.599	13.453	11.344	7.076
Cumulative percentage（%）	50.599	64.051	75.396	82.472
Feature vector				
Vicinamibacteraceae	.962	-.054	-.052	.018
Faith_pd	.951	.131	.051	-.087
Chao1	.904	.136	.275	-.194
g__Vicinamibacteraceae	.900	-.173	.047	-.049
Shannon	.894	.128	.349	-.111
g__Lysobacter	.841	-.239	-.194	.277
Pielou_e	.837	.104	.396	-.052
67-14	-.640	.366	-.061	-.012
AK	.639	.035	-.464	.297
Solirubrobacter	-.630	.428	.366	-.116
SOM	.596	.371	-.366	-.249
Nocardioides	-.530	-.408	.481	.333
g__RB41	.395	.785	.223	.020
species	.580	-.704	-.013	.015
g__Allorhizobium-Neorhizobium-Pararhizobium-Rhizobium	-.043	-.428	.647	-.399
AN	.290	.284	.478	.747

Table S18 Total Variance Explained (αBM-SQI$_{phyl}$)

	Initial Eigenvalue			Sum of Squared Loadings		
PC	Total	Percentage of Variance	Cumulative Percentage	Total	Percentage of Variance	Cumulative Percentage
1	6.279	44.849	44.849	6.279	44.849	44.849
2	2.473	17.667	62.516	2.473	17.667	62.516
3	1.388	9.915	72.431	1.388	9.915	72.431
4	1.146	8.187	80.618	1.146	8.187	80.618
5	.981	7.005	87.622			
6	.565	4.033	91.655			
7	.505	3.609	95.265			
8	.289	2.062	97.326			
9	.140	1.000	98.326			
10	.098	.700	99.026			
11	.071	.507	99.533			

12	.062	.440	99.972
13	.004	.026	99.998
14	.000	.002	100.000

Table S19 Principal Component Analysis Results (αBM-SQI_{phyl})

PCs	PC-1	PC-2	PC-3	PC-4
Eigenvalue	6.279	2.473	1.388	1.146
Variance Explanation Percentage（%）	44.849	17.667	9.915	8.187
Cumulative percentage（%）	44.849	62.516	72.431	80.618
Feature vector				
Chao1	.967	.096	-.147	.017
Faith_pd	.964	-.138	-.013	-.056
Shannon	.951	.099	-.089	.149
Pielou_e	.892	.136	-.066	.233
s__unclassified_Vicinamibacteraceae	.868	-.022	.321	-.010
uncultured_Gemmatimonadetes_bacterium	.663	.463	-.412	-.220
SOM	.561	-.435	-.181	-.362
Archangium_gephyra	-.235	.766	-.120	.039
Goods_coverage	-.540	-.571	.243	.114
AK	.555	-.559	.299	-.113
s__unclassified_TRA3-20	.397	-.476	.266	.049
Sporichthya_polymorpha	-.105	.608	.693	.044
species	.548	.432	.575	-.183
AN	.292	-.071	-.055	.906

Table S20 Total Variance Explained (αBM-SQI_{cls})

	Initial Eigenvalue			Sum of Squared Loadings		
PC	Total	Percentage of Variance	Cumulative Percentage	Total	Percentage of Variance	Cumulative Percentage
1	8.295	46.084	46.084	8.295	46.084	46.084
2	2.890	16.057	62.141	2.890	16.057	62.141
3	1.991	11.064	73.205	1.991	11.064	73.205
4	1.402	7.790	80.995	1.402	7.790	80.995
5	1.196	6.647	87.642	1.196	6.647	87.642
6	.663	3.681	91.323			
7	.464	2.579	93.901			
8	.419	2.328	96.229			
9	.197	1.097	97.326			
10	.187	1.037	98.363			
11	.144	.799	99.162			
12	.077	.428	99.590			
13	.054	.298	99.888			
14	.013	.073	99.961			

15	.005	.026	99.987
16	.002	.012	99.999
17	.000	.001	100.000
18	1.970E-5	.000	100.000

Table S21 Principal Component Analysis Results (αBM-SQI_{cls})

PCs	PC-1	PC-2	PC-3	PC-4	PC-5
Eigenvalue	8.295	2.89	1.991	1.402	1.196
Variance Explanation Percentage （%）	46.084	16.057	11.064	7.79	6.647
Cumulative percentage（%）	46.084	62.141	73.205	80.995	87.642
Feature vector					
Observed_species	.970	-.116	-.042	.025	-.113
Faith_pd	.963	.094	.067	.016	-.052
Shannon	.963	-.189	-.058	.106	.008
Chao1	.962	-.140	-.105	.017	-.111
Pielou_e	.910	-.234	-.062	.169	.087
Acidobacteriota	.859	-.022	.402	-.046	-.245
Actinobacteriota	-.833	-.109	.009	.342	-.171
Simpson	.797	-.203	-.125	.421	.074
SOM	.596	.282	.341	-.082	-.355
Na	.160	.907	-.107	-.026	-.020
EC	.290	.842	.043	.145	-.200
AK	.543	.688	.199	.160	.073
Chloroflexi	-.265	.589	-.286	.357	.399
Gemmatimonadota	.195	-.013	-.852	-.062	-.275
Goods_coverage	-.480	.253	.680	.031	.055
Proteobacteria	.469	-.067	.274	-.713	.382
AN	.294	-.375	.394	.571	.401
species	.525	.251	-.348	-.210	.590

Table S22 Total Variance Explained (αBM-SQI_{ord})

	Initial Eigenvalue			Sum of Squared Loadings		
PC	Total	Percentage of Variance	Cumulative Percentage	Total	Percentage of Variance	Cumulative Percentage
1	7.354	49.024	49.024	7.354	49.024	49.024
2	2.173	14.489	63.514	2.173	14.489	63.514
3	1.637	10.916	74.430	1.637	10.916	74.430
4	1.161	7.738	82.168	1.161	7.738	82.168
5	.893	5.952	88.119			
6	.561	3.740	91.859			
7	.415	2.769	94.628			
8	.326	2.171	96.799			

9	.270	1.799	98.598			
10	.138	.921	99.519			
11	.040	.269	99.788			
12	.029	.195	99.983			
13	.002	.016	99.999			
14	.000	.001	100.000			
15	2.950E-5	.000	100.000			

Table S23 Principal Component Analysis Results (αBM-SQI_{ord})

PCs	PC-1	PC-2	PC-3	PC-4
Eigenvalue	7.354	2.173	1.637	1.161
Variance Explanation Percentage（%）	49.024	14.489	10.916	7.738
Cumulative percentage（%）	49.024	63.514	74.43	82.168
Feature vector				
Observed_species	.959	-.063	-.167	.019
Faith_pd	.954	.119	-.044	.083
Chao1	.952	-.092	-.208	-.036
Shannon	.949	-.162	-.158	.111
Pielou_e	.894	-.223	-.158	.176
Thermoleophilia	-.782	.029	-.342	.247
Acidimicrobiia	-.693	.485	-.289	-.073
Gammaproteobacteria	.660	-.090	.379	-.346
Actinobacteria	-.592	-.445	-.051	.244
SOM	.588	.360	.252	.183
EC	.273	.889	-.101	.042
AK	.543	.654	.107	.234
Alphaproteobacteria	.084	-.250	.877	.041
AN	.286	-.363	-.191	.697
Goods_coverage	-.485	.294	.527	.535

Table S24 Total Variance Explained (αBM-SQI_{fam})

	Initial Eigenvalue			Sum of Squared Loadings		
PC	Total	Percentage of Variance	Cumulative Percentage	Total	Percentage of Variance	Cumulative Percentage
1	7.306	45.661	45.661	7.306	45.661	45.661
2	2.745	17.156	62.817	2.745	17.156	62.817
3	1.630	10.188	73.005	1.630	10.188	73.005
4	1.378	8.612	81.616	1.378	8.612	81.616
5	.850	5.315	86.932			
6	.593	3.709	90.641			
7	.502	3.135	93.776			
8	.418	2.613	96.390			

9	.237	1.484	97.874
10	.174	1.088	98.961
11	.099	.619	99.581
12	.041	.259	99.840
13	.015	.095	99.935
14	.008	.052	99.987
15	.002	.013	100.000
16	4.058E-5	.000	100.000

Table S25 Principal Component Analysis Results (αBM-SQI$_{fam}$)

PCs	PC-1	PC-2	PC-3	PC-4
Eigenvalue	7.306	2.745	1.63	1.378
Variance Explanation Percentage（%）	45.661	17.156	10.188	8.612
Cumulative percentage（%）	45.661	62.817	73.005	81.616
Feature vector				
Chao1	.948	-.125	-.064	.048
Observed_species	.947	-.097	-.010	.069
Faith_pd	.936	.105	.109	.066
Vicinamibacterales	.909	.167	.108	-.108
Pielou_e	.905	-.229	.028	.178
Solirubrobacterales	-.807	-.002	-.249	.424
Simpson	.806	-.215	-.078	.413
species	.614	.204	-.007	-.393
Goods_coverage	-.556	.290	.552	.158
Burkholderiales	.545	-.431	.347	-.371
Na	.195	.915	-.146	-.090
EC	.275	.885	-.047	.123
AK(g/kg)	.533	.678	.257	.136
Gemmatimonadales	.403	-.256	-.677	-.185
Sphingomonadales	-.252	-.111	.553	-.500
AN	.287	-.336	.504	.574

Table S26 Total Variance Explained (α-SQI)

	Initial Eigenvalue			Sum of Squared Loadings		
PC	Total	Percentage of Variance	Cumulative Percentage	Total	Percentage of Variance	Cumulative Percentage
1	8.869	46.680	46.680	8.869	46.680	46.680
2	2.813	14.805	61.485	2.813	14.805	61.485
3	1.647	8.668	70.153	1.647	8.668	70.153
4	1.440	7.576	77.730	1.440	7.576	77.730
5	1.158	6.096	83.825	1.158	6.096	83.825
6	.881	4.636	88.462			
7	.572	3.010	91.471			

8	.516	2.713	94.185
9	.451	2.371	96.556
10	.264	1.390	97.946
11	.168	.884	98.830
12	.077	.404	99.234
13	.074	.389	99.623
14	.048	.251	99.874
15	.014	.075	99.949
16	.009	.045	99.994
17	.001	.005	99.999
18	.000	.001	100.000
19	2.932E-5	.000	100.000

Table S27 Principal Component Analysis Results (α-SQI)

PCs	PC-1	PC-2	PC-3	PC-4	PC-5
Eigenvalue	8.869	2.813	1.647	1.44	1.158
Variance Explanation Percentage（%）	46.68	14.805	8.668	7.576	6.096
Cumulative percentage（%）	46.68	61.485	70.153	77.73	83.825
Feature vector					
Faith_pd	.949	.071	.102	.041	-.024
Shannon	.949	-.238	.065	.088	-.005
Observed_species	.947	-.148	.022	.092	-.074
Chao1	.943	-.183	-.035	.083	-.073
Vicinamibacteraceae	.905	.141	.016	-.085	.153
Pielou_e	.903	-.293	.093	.098	.047
Solirubrobacteraceae	-.819	-.068	.012	.437	-.019
Simpson	.808	-.290	.064	.319	.136
67-14	-.618	.046	-.155	.566	.046
species	.605	.124	-.220	-.554	.204
SOM	.584	.371	.265	.247	-.496
Na	.221	.853	-.341	-.003	.175
EC	.314	.814	-.172	.211	.168
AK	.568	.660	.138	.036	.215

Table S28 Correlation Matrix (αMA-SQI_{phyl})

		Chao1	Faith_pd	Goods_coverage	Observed_species	Pielou_e	Shannon	Simpson	pH
Correlation	Chao1	1.000	.937	-.623	.997	.954	.758	.103	.192
	Faith_pd	.937	1.000	-.408	.955	.899	.700	-.049	.341
	Goods_coverage	-.623	-.408	1.000	-.559	-.490	-.413	-.429	.085
	Observed_species	.997	.955	-.559	1.000	.959	.758	.062	.215
	Pielou_e	.880	.816	-.419	.886	.980	.917	.132	.098
	Shannon	.954	.899	-.490	.959	1.000	.875	.111	.152
	Simpson	.758	.700	-.413	.758	.875	1.000	.081	.072
	pH	.103	-.049	-.429	.062	.111	.081	1.000	.233
	EC	.192	.341	.085	.215	.152	.072	.233	1.000
	SOM	.510	.632	.048	.547	.486	.379	-.538	.345
	AN	.277	.251	-.020	.292	.367	.450	.010	-.178
	TN	-.393	-.437	.123	-.406	-.386	-.364	.271	-.252
	AP	.114	.075	-.275	.092	.023	-.035	.291	.500
	TP	.125	.249	.231	.155	.240	.301	.045	.612
	Na	.058	.221	.015	.069	-.021	-.068	.075	.820
	AK	.390	.615	.033	.424	.377	.317	-.062	.734

		EC	SOM	AN	TN	AP	TP	Na	AK
Correlation	Chao1	.192	.510	.277	-.393	.114	.125	.058	.390
	Faith_pd	.341	.632	.251	-.437	.075	.249	.221	.615
	Goods_coverage	.085	.048	-.020	.123	-.275	.231	.015	.033
	Observed_species	.215	.547	.292	-.406	.092	.155	.069	.424

Continued table S28 CorrelationMatrix（αMA-SQI_{phyl}）

		EC	SOM	AN	TN	AP	TP	Na	AK
Correlation	Pielou_e	.098	.427	.413	-.353	-.022	.286	-.080	.319
	Shannon	.152	.486	.367	-.386	.023	.240	-.021	.377
	Simpson	.072	.379	.450	-.364	-.035	.301	-.068	.317
	pH	.233	-.538	.010	.271	.291	.045	.075	-.062
	EC	1.000	.345	-.178	-.252	.500	.612	.820	.734
	SOM	.345	1.000	-.046	-.668	.064	.275	.270	.459
	AN	-.178	-.046	1.000	.012	-.193	.166	-.301	.142
	TN	-.252	-.668	.012	1.000	-.170	-.347	-.226	-.397
	AP	.500	.064	-.193	-.170	1.000	.311	.490	.328
	TP	.612	.275	.166	-.347	.311	1.000	.521	.678

Na	.820	.270	-.301	-.226	.490	.521	1.000	.649
AK	.734	.459	.142	-.397	.328	.678	.649	1.000

Table S29 Correlation Matrix (αMA-SQI_{cls})

		AN	AK	Actinobacteriota	p__Acidobacteriota	Chao1	SOM	EC	Faith_pd	Pielou_e
Correlation	AN	1.000	.142	-.086	.259	.277	-.046	-.178	.251	.413
	AK	.142	1.000	-.508	.374	.390	.459	.734	.615	.319
	Actinobacteriota	-.086	-.508	1.000	-.751	-.728	-.501	-.231	-.762	-.680
	p__Acidobacteriota	.259	.374	-.751	1.000	.817	.535	.215	.786	.686
	Chao1	.277	.390	-.728	.817	1.000	.510	.192	.937	.880
	SOM	-.046	.459	-.501	.535	.510	1.000	.345	.632	.427
	EC	-.178	.734	-.231	.215	.192	.345	1.000	.341	.098
	Faith_pd	.251	.615	-.762	.786	.937	.632	.341	1.000	.816

Continued table S29 CorrelationMatrix（αMA-SQI_{cls}）

		AN	AK	Actinobacteriota	p__Acidobacteriota	Chao1	SOM	EC	Faith_pd	Pielou_e
Correlation	Pielou_e	.413	.319	-.680	.686	.880	.427	.098	.816	1.000
	p__Actinobacteriota	-.219	-.567	.918	-.711	-.791	-.560	-.180	-.865	-.788
	Chloroflexi	-.100	.259	.118	-.519	-.349	-.103	.292	-.213	-.287
	Gemmatimonadota	-.379	-.031	-.197	-.034	.271	-.071	.047	.121	.230
	Proteobacteria	.056	.214	-.712	.448	.373	.247	-.064	.414	.339
	Acidobacteriota	.307	.511	-.718	.938	.819	.687	.300	.872	.706
	p__Proteobacteria	-.024	-.117	-.527	.269	.114	-.117	-.263	.030	.100
	Shannon	.367	.377	-.725	.761	.954	.486	.152	.899	.980
	p__Chloroflexi	-.208	-.075	.307	-.612	-.537	-.322	.111	-.505	-.498

		p__Actinobacteriota	Chloroflexi	Gemmatimonadota	Proteobacteria	Acidobacteriota	p__Proteobacteria	Shannon	p__Chloroflexi
Correlation	AN	-.219	-.100	-.379	.056	.307	-.024	.367	-.208
	AK	-.567	.259	-.031	.214	.511	-.117	.377	-.075
	Actinobacteriota	.918	.118	-.197	-.712	-.718	-.527	-.725	.307
	p__Acidobacteriota	-.711	-.519	-.034	.448	.938	.269	.761	-.612
	Chao1	-.791	-.349	.271	.373	.819	.114	.954	-.537
	SOM	-.560	-.103	-.071	.247	.687	-.117	.486	-.322
	EC	-.180	.292	.047	-.064	.300	-.263	.152	.111
	Faith_pd	-.865	-.213	.121	.414	.872	.030	.899	-.505
	Pielou_e	-.788	-.287	.230	.339	.706	.100	.980	-.498
	p__Actinobacteriota	1.000	.195	-.154	-.688	-.780	-.345	-.827	.489
	Chloroflexi	.195	1.000	.064	-.344	-.460	-.268	-.322	.905

Continued table S29 CorrelationMatrix（αMA-SQI$_{cls}$）

		p__Actino bacteriota	Chloroflexi	Gemmatimonadota	Proteobacteria	Acido bacteriota	p__Proteo bacteria	Shannon	p__Chloro flexi
Correlation	Gemmatimonadota	-.154	.064	1.000	-.152	-.120	-.064	.240	.083
	Proteobacteria	-.688	-.344	-.152	1.000	.446	.828	.376	-.458
	Acidobacteriota	-.780	-.460	-.120	.446	1.000	.092	.784	-.667
	p__Proteobacteria	-.345	-.268	-.064	.828	.092	1.000	.105	-.164
	Shannon	-.827	-.322	.240	.376	.784	.105	1.000	-.541
	p__Chloroflexi	.489	.905	.083	-.458	-.667	-.164	-.541	1.000

Table S30 Correlation Matrix (αMA-SQI$_{ord}$)

		SOM	AN	AK	species	Chao1	Actinobacteria	Thermoleophilia	Vicinamibacteria
Correla	SOM	1.000	-.046	.459	.134	.510	-.430	-.422	.400

AN	-.046	1.000	.142	-.015	.277	.070	-.090	.138
AK	.459	.142	1.000	.324	.390	-.462	-.507	.352
species	.134	-.015	.324	1.000	.443	-.109	-.740	.554
Chao1	.510	.277	.390	.443	1.000	-.514	-.624	.793
Actinobacteria	-.430	.070	-.462	-.109	-.514	1.000	.385	-.587
Thermoleophilia	-.422	-.090	-.507	-.740	-.624	.385	1.000	-.741
c__Vicinamibacteria	.400	.138	.352	.554	.793	-.587	-.741	1.000
c__Gammaproteobacteria	.010	.088	.083	.519	.373	-.346	-.737	.749
c__Blastocatellia	.445	.458	.215	-.387	.523	-.390	-.128	.238
c__Alphaproteobacteria	-.265	-.196	-.385	-.108	-.368	.084	.016	-.301
pH	-.538	.010	-.062	.330	.103	-.013	.002	.233
Gemmatimonadetes	-.001	-.196	-.065	.157	.438	-.120	-.171	.338

Continued table S30 CorrelationMatrix（αMA-SQI$_{ord}$）

		SOM	AN	AK	species	Chao1	Actinobacteria	Thermoleophilia	Vicinamibacteria
Correlation	Gammaproteobacteria	.232	.112	.308	.599	.522	-.434	-.871	.804
	Shannon	.486	.367	.377	.486	.954	-.446	-.629	.753

		Gammaproteobacteria	Blastocatellia	Alphaproteobacteria	Gemmatimonadetes	Gammaproteobacteria	Shannon
	SOM	.010	.445	-.265	-.001	.232	.486
	AN	.088	.458	-.196	-.196	.112	.367
	AK	.083	.215	-.385	-.065	.308	.377
	species	.519	-.387	-.108	.157	.599	.486
	Chao1	.373	.523	-.368	.438	.522	.954
	Actinobacteria	-.346	-.390	.084	-.120	-.434	-.446
	Thermoleophilia	-.737	-.128	.016	-.171	-.871	-.629
Correlation	c__Vicinamibacteria	.749	.238	-.301	.338	.804	.753
	c__Gammaproteobacteria	1.000	-.022	.263	.160	.929	.365
	c__Blastocatellia	-.022	1.000	-.140	.015	.119	.486
	c__Alphaproteobacteria	.263	-.140	1.000	-.182	.157	-.374
	pH	.279	-.406	-.081	.267	.150	.111
	Gemmatimonadetes	.160	.015	-.182	.267	.446	.828
	Gammaproteobacteria	.929	.119	.157	.150	1.000	.092
	Shannon	.365	.486	-.374	.111	.092	1.000

Table S31 Correlation Matrix (αMA-SQI$_{fam}$)

		SOM	AN	AK	species	Gemmatimonadales	Solirubrobacterales	Vicinamibacterales	Burkholderiales
Correlation	SOM	1.000	-.046	.459	.134	-.001	-.408	.576	.132
	AN	-.046	1.000	.142	-.015	-.196	-.150	.176	.222
	AK	.459	.142	1.000	.324	-.065	-.463	.535	.099
	species	.134	-.015	.324	1.000	.157	-.753	.565	.241
	Gemmatimonadales	-.001	-.196	-.065	.157	1.000	-.233	.248	.140
	Solirubrobacterales	-.408	-.150	-.463	-.753	-.233	1.000	-.791	-.698
	Vicinamibacterales	.576	.176	.535	.565	.248	-.791	1.000	.550
	Burkholderiales	.132	.222	.099	.241	.140	-.698	.550	1.000
	o__Vicinamibacterales	.389	.136	.351	.569	.347	-.749	.935	.609
	o__Xanthomonadales	.222	.136	.286	.647	.182	-.794	.792	.619
	o__Pyrinomonadales	.416	.451	.207	-.417	.024	-.086	.302	.356
	Shannon	.486	.367	.377	.486	.431	-.680	.815	.489
	Simpson	.379	.450	.317	.383	.352	-.492	.598	.358
	Faith_pd	.632	.251	.615	.490	.241	-.706	.868	.442
	Pielou_e	.427	.413	.319	.492	.428	-.656	.753	.471

		o__Vicinamibacterales	o__Xanthomonadales	o__Pyrinomonadales	Shannon	Simpson	Faith_pd	Pielou_e
Correlation	SOM	.389	.222	.416	.486	.379	.632	.427
	AN	.136	.136	.451	.367	.450	.251	.413
	AK	.351	.286	.207	.377	.317	.615	.319

Continued table S31 CorrelationMatrix（αMA-SQI$_{fam}$）

		o__Vicinamibacterales	o__Xanthomonadales	o__Pyrinomonadales	Shannon	Simpson	Faith_pd	Pielou_e
Correlation	species	.569	.647	-.417	.486	.383	.490	.492
	Gemmatimonadales	.347	.182	.024	.431	.352	.241	.428
	Solirubrobacterales	-.749	-.794	-.086	-.680	-.492	-.706	-.656
	Vicinamibacterales	.935	.792	.302	.815	.598	.868	.753
	Burkholderiales	.609	.619	.356	.489	.358	.442	.471
	o__Vicinamibacterales	1.000	.882	.159	.744	.568	.718	.694
	o__Xanthomonadales	.882	1.000	-.038	.546	.366	.502	.524
	o__Pyrinomonadales	.159	-.038	1.000	.456	.341	.495	.389

	Shannon	.744	.546	.456	1.000	.875	.899	.980
	Simpson	.568	.366	.341	.875	1.000	.700	.917
	Faith_pd	.718	.502	.495	.899	.700	1.000	.816
	Pielou_e	.694	.524	.389	.980	.917	.816	1.000

Table S32 Correlation Matrix (αMA-SQI_{gen})

		EC	SOM	AN	AK	species	Chao1	Gemmatimonadaceae	Vicinamibacteraceae	Solirubrobacteraceae	Vicinamibacteraceae
Correlation	EC	1.000	.345	-.178	.734	.196	.192	-.040	.431	-.220	.304
	SOM	.345	1.000	-.046	.459	.134	.510	-.001	.514	-.429	.350
	AN	-.178	-.046	1.000	.142	-.015	.277	-.196	.241	-.133	.210
	AK	.734	.459	.142	1.000	.324	.390	-.065	.596	-.437	.426
	species	.196	.134	-.015	.324	1.000	.443	.157	.560	-.751	.573
	Chao1	.192	.510	.277	.390	.443	1.000	.438	.837	-.688	.800

Continued table S32 CorrelationMatrix（αMA-SQI_{gen}）

		EC	SOM	AN	AK	species	Chao1	Gemmatimonadaceae	Vicinamibacteraceae	Solirubrobacteraceae	Vicinamibacteraceae
Correlation	Gemmatimonadaceae	-.040	-.001	-.196	-.065	.157	.438	1.000	.215	-.299	.315
	Vicinamibacteraceae	.431	.514	.241	.596	.560	.837	.215	1.000	-.840	.944
	Solirubrobacteraceae	-.220	-.429	-.133	-.437	-.751	-.688	-.299	-.840	1.000	-.829
	f__Vicinamibacteraceae	.304	.350	.210	.426	.573	.800	.315	.944	-.829	1.000
	f__Xanthomonadaceae	.429	.415	.118	.588	.627	.627	.116	.857	-.883	.848
	f__Pyrinomonadaceae	-.054	.416	.451	.207	-.417	.492	.024	.325	-.074	.201
	f__Rhizobiaceae	-.195	-.031	-.099	-.101	.345	.202	.264	.168	-.423	.255
	Faith_pd	.341	.632	.251	.615	.490	.937	.241	.879	-.703	.754
	Goods_coverage	.085	.048	-.020	.033	-.349	-.623	-.589	-.388	.331	-.570
	Pielou_e	.098	.427	.413	.319	.492	.880	.428	.751	-.704	.713
	Shannon	.152	.486	.367	.377	.486	.954	.431	.813	-.721	.764
	Simpson	.072	.379	.450	.317	.383	.758	.352	.613	-.520	.599
	Nocardioidaceae	-.395	-.389	.158	-.304	.104	-.288	-.134	-.272	.148	-.269

		Xanthomonadaceae	Pyrinomonadaceae	Rhizobiaceae	Faith_pd	Goods_coverage	Pielou_e	Shannon	Simpson	Nocardioidaceae
Correlation	EC	.429	-.054	-.195	.341	.085	.098	.152	.072	-.395
	SOM	.415	.416	-.031	.632	.048	.427	.486	.379	-.389
	AN	.118	.451	-.099	.251	-.020	.413	.367	.450	.158
	AK	.588	.207	-.101	.615	.033	.319	.377	.317	-.304
	species	.627	-.417	.345	.490	-.349	.492	.486	.383	.104
	Chao1	.627	.492	.202	.937	-.623	.880	.954	.758	-.288

Continued table S32 CorrelationMatrix（αMA-SQI$_{gen}$）

		Xanthomonadaceae	Pyrinomonadaceae	Rhizobiaceae	Faith_pd	Goods_coverage	Pielou_e	Shannon	Simpson	Nocardioidaceae
Correlation	Gemmatimonadaceae	.116	.024	.264	.241	-.589	.428	.431	.352	-.134
	Vicinamibacteraceae	.857	.325	.168	.879	-.388	.751	.813	.613	-.272
	Solirubrobacteraceae	-.883	-.074	-.423	-.703	.331	-.704	-.721	-.520	.148
	f__Vicinamibacteraceae	.848	.201	.255	.426	.573	.800	.315	.944	-.269
	f__Xanthomonadaceae	1.000	.090	.277	.588	.627	.627	.116	.857	-.325
	f__Pyrinomonadaceae	.090	1.000	-.106	.207	-.417	.492	.024	.325	-.275
	f__Rhizobiaceae	.277	-.106	1.000	-.101	.345	.202	.264	.168	.309
	Faith_pd	.678	.495	.132	.615	.490	.937	.241	.879	-.290
	Goods_coverage	-.316	-.005	-.231	.033	-.349	-.623	-.589	-.388	.150
	Pielou_e	.098	.427	.413	.319	.492	.880	.428	.751	-.147
	Shannon	.152	.486	.367	.377	.486	.954	.431	.813	-.205
	Simpson	.072	.379	.450	.317	.383	.758	.352	.613	-.165
	Nocardioidaceae	-.395	-.389	.158	-.304	.104	-.288	-.134	-.272	1.000

Table S33 Correlation Matrix (αMA-SQI$_{spp}$)

		SOM	AN	AK	species	Vicinamibacteraceae	67-14	g__Vicinamibacteraceae	g__Lysobacter
Correlation	SOM	1.000	-.046	.459	.134	.533	-.303	.376	.348
	AN	-.046	1.000	.142	-.015	.242	-.143	.213	.268
	AK	.459	.142	1.000	.324	.611	-.419	.452	.708

		SOM	AN	AK	species	Vicinamibacteraceae	67-14	g__Vicinamibacteraceae	g__Lysobacter
	species	.134	-.015	.324	1.000	.541	-.602	.560	.612
	Vicinamibacteraceae	.533	.242	.611	.541	1.000	-.558	.946	.876

Continued table S33 CorrelationMatrix（αMA-SQI_{spp}）

		SOM	AN	AK	species	Vicinamibacteraceae	67-14	g__Vicinamibacteraceae	g__Lysobacter
Correlation	67-14	-.303	-.143	-.419	-.602	-.558	1.000	-.538	-.450
	g__Vicinamibacteraceae	.376	.213	.452	.560	.946	-.538	1.000	.847
	g__Lysobacter	.348	.268	.708	.612	.876	-.450	.847	1.000
	g__RB41	.416	.451	.207	-.417	.351	-.098	.234	.084
	g__Allorhizobium-Neorhizobium-Pararhizobium-Rhizobium	-.339	-.120	-.322	.124	-.027	-.176	.119	-.122
	Chao1	.510	.277	.390	.443	.835	-.488	.808	.635
	Nocardioides	-.545	.169	-.441	-.023	-.504	.134	-.478	-.376
	Solirubrobacter	-.343	-.005	-.477	-.566	-.690	.577	-.662	-.720
	Faith_pd	.632	.251	.615	.490	.886	-.581	.774	.706
	Shannon	.486	.367	.377	.486	.811	-.499	.772	.624
	Pielou_e	.427	.413	.319	.492	.747	-.471	.718	.584

		g__RB41	g__Allorhizobium-Neorhizobium-Pararhizobium-Rhizobium	Chao1	Nocardioides	Solirubrobacter	Faith_pd	Shannon	Pielou_e
Correlation	SOM	.416	-.339	.510	-.545	-.343	.632	.486	.427
	AN	.451	-.120	.277	.169	-.005	.251	.367	.413
	AK	.207	-.322	.390	-.441	-.477	.615	.377	.319
	species	-.417	.124	.443	-.023	-.566	.490	.486	.492
	Vicinamibacteraceae	.351	-.027	.835	-.504	-.690	.886	.811	.747

Continued table S33 CorrelationMatrix（αMA-SQI_{spp}）

		g__RB41	g__Allorhizobium-Neorhizobium-Pararhizobium-Rhizobium	Chao1	Nocardioides	Solirubrobacter	Faith_pd	Shannon	Pielou_e

Correlation	67-14	-.098	-.176	-.488	.134	.577	-.581	-.499	-.471
	g__Vicinamibacteraceae	.234	.119	.808	-.478	-.662	.774	.772	.718
	g__Lysobacter	.084	-.122	.635	-.376	-.720	.706	.624	.584
	g__RB41	1.000	-.093	.492	-.395	.011	.495	.456	.389
	g__Allorhizobium-Neorhizobium-Pararhizobium-Rhizobium	-.093	1.000	.125	.377	.032	-.038	.098	.082
	Chao1	.492	.125	1.000	-.464	-.366	.937	.954	.880
	Nocardioides	-.395	.377	-.464	1.000	.279	-.510	-.395	-.322
	Solirubrobacter	.011	.032	-.366	.279	1.000	-.489	-.295	-.231
	Faith_pd	.495	-.038	.937	-.510	-.489	1.000	.899	.816
	Shannon	.456	.098	.954	-.395	-.295	.899	1.000	.980
	Pielou_e	.389	.082	.880	-.322	-.231	.816	.980	1.000

Table S34 Correlation Matrix (αBM-SQI$_{phyl}$)

		SOM	AN	AK	species	Sporichthya_polymorpha	uncultured_Gemmatimonadetes_bacterium	s__unclassified_Vicinamibacteraceae
Correlation	SOM	1.000	-.046	.459	.134	-.380	.246	.445
	AN	-.046	1.000	.142	-.015	-.069	.013	.237
	AK	.459	.142	1.000	.324	-.303	.056	.557
	species	.134	-.015	.324	1.000	.521	.364	.568
	Sporichthya_polymorpha	-.380	-.069	-.303	.521	1.000	-.061	.137
	uncultured_Gemmatimonadetes_bacterium	.246	.013	.056	.364	-.061	1.000	.435

s__unclassified_Vicinamibacteraceae	.445	.237	.557	.568	.137	.435	1.000
Archangium_gephyra	-.398	-.098	-.477	.166	.284	.126	-.267
Chao1	.510	.277	.390	.443	-.108	.724	.792
Faith_pd	.632	.251	.615	.490	-.185	.591	.807
Goods_coverage	.048	-.020	.033	-.349	-.063	-.753	-.453
Pielou_e	.427	.413	.319	.492	-.044	.589	.689
Shannon	.486	.367	.377	.486	-.076	.646	.751
s__unclassified_TRA3-20	.115	.039	.487	.030	-.226	-.111	.476

		Archangium_gephyra	Chao1	Faith_pd	Goods_coverage	Pielou_e	Shannon	s__unclassified_TRA3-20
Correlation	SOM	-.398	.510	.632	.048	.427	.486	.115
	AN	-.098	.277	.251	-.020	.413	.367	.039
	AK	-.477	.390	.615	.033	.319	.377	.487

Continued table S34 CorrelationMatrix（αBM-SQI$_{phyl}$）

		Archangium_gephyra	Chao1	Faith_pd	Goods_coverage	Pielou_e	Shannon	s__unclassified_TRA3-20
Correlation	species	.166	.443	.490	-.349	.492	.486	.030
	Sporichthya_polymorpha	.284	-.108	-.185	-.063	-.044	-.076	-.226
	uncultured_Gemmatimonadetes_bacterium	.126	.724	.591	-.753	.589	.646	-.111
	s__unclassified_Vicinamibacteraceae	-.267	.792	.807	-.453	.689	.751	.476
	Archangium_gephyra	1.000	-.169	-.347	-.293	-.036	-.111	-.399
	Chao1	-.169	1.000	.937	-.623	.880	.954	.322
	Faith_pd	-.347	.937	1.000	-.408	.816	.899	.374
	Goods_coverage	-.293	-.623	-.408	1.000	-.419	-.490	-.121
	Pielou_e	-.036	.880	.816	-.419	1.000	.980	.273
	Shannon	-.111	.954	.899	-.490	.980	1.000	.309
	s__unclassified_TRA3-20	-.399	.322	.374	-.121	.273	.309	1.000

Table S35 Correlation Matrix (αBM-SQI_{cls})

		EC	SOM	AN	AK	species	Chao1	Faith_pd	Goods_ coverage	Pielou_e
Correlation	EC	1.000	.345	-.178	.734	.196	.192	.341	.085	.098
	SOM	.345	1.000	-.046	.459	.134	.510	.632	.048	.427
	AN	-.178	-.046	1.000	.142	-.015	.277	.251	-.020	.413
	AK	.734	.459	.142	1.000	.324	.390	.615	.033	.319
	species	.196	.134	-.015	.324	1.000	.443	.490	-.349	.492
	Chao1	.192	.510	.277	.390	.443	1.000	.937	-.623	.880
	Faith_pd	.341	.632	.251	.615	.490	.937	1.000	-.408	.816
	Goods_coverage	.085	.048	-.020	.033	-.349	-.623	-.408	1.000	-.419
	Pielou_e	.098	.427	.413	.319	.492	.880	.816	-.419	1.000
	Shannon	.152	.486	.367	.377	.486	.954	.899	-.490	.980
	Simpson	.072	.379	.450	.317	.383	.758	.700	-.413	.917
	Actinobacteriota	-.231	-.501	-.086	-.508	-.531	-.728	-.762	.384	-.680
	Proteobacteria	-.064	.247	.056	.214	.452	.373	.414	-.058	.339
	Acidobacteriota	.300	.687	.307	.511	.146	.819	.872	-.200	.706
	Gemmatimonadota	.047	-.071	-.379	-.031	.188	.271	.121	-.584	.230
	Chloroflexi	.292	-.103	-.100	.259	.210	-.349	-.213	.130	-.287
	Na	.820	.270	-.301	.649	.370	.058	.221	.015	-.080
	Observed_species	.215	.547	.292	.424	.433	.997	.955	-.559	.886

Continued table S35 CorrelationMatrix（αBM-SQI_{cls}）

		Shannon	Simpson	Actinobacteriota	Proteobacteria	Acidobacteriota	Gemmatimonadota	Chloroflexi	Na	Observed_species
Correlation	EC	.152	.072	-.231	-.064	.300	.047	.292	.820	.215
	SOM	.486	.379	-.501	.247	.687	-.071	-.103	.270	.547
	AN	.367	.450	-.086	.056	.307	-.379	-.100	-.301	.292

AK	.377	.317	-.508	.214	.511	-.031	.259	.649	.424
species	.486	.383	-.531	.452	.146	.188	.210	.370	.433
Chao1	.954	.758	-.728	.373	.819	.271	-.349	.058	.997
Faith_pd	.899	.700	-.762	.414	.872	.121	-.213	.221	.955
Goods_coverage	-.490	-.413	.384	-.058	-.200	-.584	.130	.015	-.559
Pielou_e	.980	.917	-.680	.339	.706	.230	-.287	-.080	.886
Shannon	1.000	.875	-.725	.376	.784	.240	-.322	-.021	.959
Simpson	.875	1.000	-.546	.074	.583	.231	-.102	-.068	.758
Actinobacteriota	-.725	-.546	1.000	-.712	-.718	-.197	.118	-.192	-.730
Proteobacteria	.376	.074	-.712	1.000	.446	-.152	-.344	-.012	.386
Acidobacteriota	.784	.583	-.718	.446	1.000	-.120	-.460	.081	.847
Gemmatimonadota	.240	.231	-.197	-.152	-.120	1.000	.064	.070	.227
Chloroflexi	-.322	-.102	.118	-.344	-.460	.064	1.000	.395	-.352
Na	-.021	-.068	-.192	-.012	.081	.070	.395	1.000	.069
Observed_species	.959	.758	-.730	.386	.847	.227	-.352	.069	1.000

Table S36 Correlation Matrix (αBM-SQI$_{ord}$)

		EC	SOM	AN	AK	Chao1	Faith_pd	Goods_coverage	Observed_species
	EC	1.000	.345	-.178	.734	.192	.341	.085	.215
	SOM	.345	1.000	-.046	.459	.510	.632	.048	.547
	AN	-.178	-.046	1.000	.142	.277	.251	-.020	.292
	AK	.734	.459	.142	1.000	.390	.615	.033	.424
	Chao1	.192	.510	.277	.390	1.000	.937	-.623	.997
Correlation	Faith_pd	.341	.632	.251	.615	.937	1.000	-.408	.955
	Goods_coverage	.085	.048	-.020	.033	-.623	-.408	1.000	-.559
	Pielou_e	.098	.427	.413	.319	.880	.816	-.419	.886
	Shannon	.152	.486	.367	.377	.954	.899	-.490	.959
	Actinobacteria	-.516	-.430	.070	-.462	-.514	-.534	.233	-.518
	Thermoleophilia	-.126	-.422	-.090	-.507	-.624	-.704	.343	-.626

Alphaproteobacteria	-.243	.154	-.054	-.025	-.024	.047	.306	.007
Gammaproteobacteria	.077	.232	.112	.308	.522	.530	-.285	.520
Acidimicrobiia	.221	-.320	-.294	-.116	-.618	-.548	.313	-.619
Observed_species	.215	.547	.292	.424	.997	.955	-.559	1.000

		Pielou_e	Shannon	Actino bacteria	Thermo leophilia	Alphaproteo bacteria	Gammaproteo bacteria	Acidimic robiia
Correlation	EC	.098	.152	-.516	-.126	-.243	.077	.221
	SOM	.427	.486	-.430	-.422	.154	.232	-.320
	AN	.413	.367	.070	-.090	-.054	.112	-.294

Continued table S36 CorrelationMatrix（αBM-SQI$_{ord}$)

		Pielou_e	Shannon	Actino bacteria	Thermo leophilia	Alphaproteo bacteria	Gammaproteo bacteria	Acidimic robiia
Correlation	AK	.319	.377	-.462	-.507	-.025	.308	-.116
	Chao1	.880	.954	-.514	-.624	-.024	.522	-.618
	Faith_pd	.816	.899	-.534	-.704	.047	.530	-.548
	Goods_coverage	-.419	-.490	.233	.343	.306	-.285	.313
	Pielou_e	1.000	.980	-.378	-.594	-.033	.483	-.732
	Shannon	.980	1.000	-.446	-.629	-.007	.516	-.712
	Actinobacteria	-.378	-.446	1.000	.385	-.016	-.434	.253
	Thermoleophilia	-.594	-.629	.385	1.000	-.236	-.871	.584
	Alphaproteobacteria	-.033	-.007	-.016	-.236	1.000	.290	-.443
	Gammaproteobacteria	.483	.516	-.434	-.871	.290	1.000	-.455
	Acidimicrobiia	-.732	-.712	.253	.584	-.443	-.455	1.000
	Observed_species	.886	.959	-.518	-.626	.007	.520	-.619

Table S37 Correlation Matrix (αBM-SQI$_{fam}$)

		AN	AK	Gemmati monadales	Solirubro bacterales	Vicinami bacterales	Burkhol deriales	Sphingo monadales	EC
Correlation	AN	1.000	.142	-.196	-.150	.176	.222	-.018	-.178
	AK	.142	1.000	-.065	-.463	.535	.099	-.126	.734
	Gemmatimonadales	-.196	-.065	1.000	-.233	.248	.140	-.195	-.040
	Solirubrobacterales	-.150	-.463	-.233	1.000	-.791	-.698	-.020	-.095
	Vicinamibacterales	.176	.535	.248	-.791	1.000	.550	-.187	.414

Continued table S37 CorrelationMatrix（αBM-SQI$_{fam}$）

		AN	AK	Gemmati monadales	Solirubro bacterales	Vicinami bacterales	Burkhol deriales	Sphingo monadales	EC
Correlation	Burkholderiales	.222	.099	.140	-.698	.550	1.000	.121	-.293
	Sphingomonadales	-.018	-.126	-.195	-.020	-.187	.121	1.000	-.123
	EC	-.178	.734	-.040	-.095	.414	-.293	-.123	1.000
	species	-.015	.324	.157	-.753	.565	.241	-.100	.196
	Observed_species	.292	.424	.401	-.658	.849	.477	-.159	.215
	Faith_pd	.251	.615	.241	-.706	.868	.442	-.177	.341
	Goods_coverage	-.020	.033	-.589	.347	-.384	-.312	.306	.085
	Na	-.301	.649	-.061	-.176	.321	-.263	-.225	.820
	Chao1	.277	.390	.438	-.655	.840	.484	-.179	.192
	Pielou_e	.413	.319	.428	-.656	.753	.471	-.209	.098
	Simpson	.450	.317	.352	-.492	.598	.358	-.457	.072

		species	Observed_ species	Faith_pd	Goods_ coverage	Na	Chao1	Pielou_e	Simpson
Correlation	EC	-.015	.292	.251	-.020	-.301	.277	.413	.450
	SOM	.324	.424	.615	.033	.649	.390	.319	.317
	AN	.157	.401	.241	-.589	-.061	.438	.428	.352
	AK	-.753	-.658	-.706	.347	-.176	-.655	-.656	-.492
	species	.565	.849	.868	-.384	.321	.840	.753	.598
	Chao1	.241	.477	.442	-.312	-.263	.484	.471	.358
	Faith_pd	-.100	-.159	-.177	.306	-.225	-.179	-.209	-.457
	Goods_coverage	.196	.215	.341	.085	.820	.192	.098	.072

Continued table S37 CorrelationMatrix（αBM-SQI$_{fam}$）

		species	Observed_ species	Faith_pd	Goods_ coverage	Na	Chao1	Pielou_e	Simpson
Correlation	Pielou_e	1.000	.433	.490	-.349	.370	.443	.492	.383
	Shannon	.433	1.000	.955	-.559	.069	.997	.886	.758
	Simpson	.490	.955	1.000	-.408	.221	.937	.816	.700
	Actinobacteriota	-.349	-.559	-.408	1.000	.015	-.623	-.419	-.413
	Proteobacteria	.370	.069	.221	.015	1.000	.058	-.080	-.068
	Acidobacteriota	.443	.997	.937	-.623	.058	1.000	.880	.758
	Gemmatimonadota	.492	.886	.816	-.419	-.080	.880	1.000	.917
	Chloroflexi	.383	.758	.700	-.413	-.068	.758	.917	1.000

Table S38 Correlation Matrix (α-SQI)

		EC	SOM	AN	AK	species	Chao1	Faith_pd	Goods_ coverage	Observed _species
Correlation	EC	1.000	.345	-.178	.734	.196	.192	.341	.085	.215

	SOM	.345	1.000	-.046	.459	.134	.510	.632	.048	.547
	AN	-.178	-.046	1.000	.142	-.015	.277	.251	-.020	.292
	AK	.734	.459	.142	1.000	.324	.390	.615	.033	.424
	species	.196	.134	-.015	.324	1.000	.443	.490	-.349	.433
	Chao1	.192	.510	.277	.390	.443	1.000	.937	-.623	.997
	Faith_pd	.341	.632	.251	.615	.490	.937	1.000	-.408	.955
	Goods_coverage	.085	.048	-.020	.033	-.349	-.623	-.408	1.000	-.559
	Observed_species	.215	.547	.292	.424	.433	.997	.955	-.559	1.000
	Pielou_e	.098	.427	.413	.319	.492	.880	.816	-.419	.886

Continued table S38 CorrelationMatrix（α-SQI）

		EC	SOM	AN	AK	species	Chao1	Faith_pd	Goods_coverage	Observed_species
	Shannon	.152	.486	.367	.377	.486	.954	.899	-.490	.959
	Simpson	.072	.379	.450	.317	.383	.758	.700	-.413	.758
	Gemmatimonadaceae	-.040	-.001	-.196	-.065	.157	.438	.241	-.589	.401
	Vicinamibacteraceae	.431	.514	.241	.596	.560	.837	.879	-.388	.847
Correlation	Solirubrobacteraceae	-.220	-.429	-.133	-.437	-.751	-.688	-.703	.331	-.691
	Sphingomonadaceae	-.123	-.164	-.018	-.126	-.100	-.179	-.177	.306	-.159
	67-14	.103	-.303	-.143	-.419	-.602	-.488	-.581	.302	-.489
	TN	-.252	-.668	.012	-.397	-.202	-.393	-.437	.123	-.406
	Na	.820	.270	-.301	.649	.370	.058	.221	.015	.069

		Pielou_e	Shannon	Simpson	Gemmatimonadaceae	Vicinamibacteraceae	Solirubrobacteraceae	Sphingomonadaceae	67-14	TN	Na
	EC	.098	.152	.072	-.040	.431	-.220	-.123	.103	-.252	.820
	SOM	.427	.486	.379	-.001	.514	-.429	-.164	-.303	-.668	.270
	AN	.413	.367	.450	-.196	.241	-.133	-.018	-.143	.012	-.301
Correlation	AK	.319	.377	.317	-.065	.596	-.437	-.126	-.419	-.397	.649
	species	.492	.486	.383	.157	.560	-.751	-.100	-.602	-.202	.370
	Chao1	.880	.954	.758	.438	.837	-.688	-.179	-.488	-.393	.058
	Faith_pd	.816	.899	.700	.241	.879	-.703	-.177	-.581	-.437	.221

Goods_coverage	-.419	-.490	-.413	-.589	-.388	.331	.306	.302	.123	.015
Observed_species	.886	.959	.758	.401	.847	-.691	-.159	-.489	-.406	.069
Pielou_e	1.000	.980	.917	.428	.751	-.704	-.209	-.471	-.353	-.080

Continued table S38 CorrelationMatrix（α-SQI）

		Pielou_e	Shannon	Simpson	Gemmatimonadaceae	Vicinamibacteraceae	Solirubrobacteraceae	Sphingomonadaceae	67-14	TN	Na
	Shannon	.980	1.000	.875	.431	.813	-.721	-.183	-.499	-.386	-.021
	Simpson	.917	.875	1.000	.352	.613	-.520	-.457	-.369	-.364	-.068
	Gemmatimonadaceae	.428	.431	.352	1.000	.215	-.299	-.195	-.107	.002	-.061
	Vicinamibacteraceae	.751	.813	.613	.215	1.000	-.840	-.199	-.561	-.304	.305
Correlation	Solirubrobacteraceae	-.704	-.721	-.520	-.299	-.840	1.000	-.009	.685	.334	-.255
	Sphingomonadaceae	-.209	-.183	-.457	-.195	-.199	-.009	1.000	-.030	.036	-.225
	67-14	-.471	-.499	-.369	-.107	-.561	.685	-.030	1.000	.311	-.018
	TN	-.353	-.386	-.364	.002	-.304	.334	.036	.311	1.000	-.226
	Na	-.080	-.021	-.068	-.061	.305	-.255	-.225	-.018	-.226	1.000
